# The prevalence of onchocerciasis in Africa and Yemen, 2000–2018: a geospatial analysis

**DOI:** 10.1186/s12916-022-02486-y

**Published:** 2022-09-07

**Authors:** Chris A. Schmidt, Elizabeth A. Cromwell, Elex Hill, Katie M. Donkers, Megan F. Schipp, Kimberly B. Johnson, David M. Pigott, LBD 2019 Neglected Tropical Diseases Collaborators, Chris A. Schmidt, Elizabeth A. Cromwell, Elex Hill, David M. Pigott, Jaffar Abbas, Victor Adekanmbi, Olatunji O. Adetokunboh, Muktar Beshir Ahmed, Fahad Mashhour Alanezi, Turki M. Alanzi, Vahid Alipour, Catalina Liliana Andrei, Tudorel Andrei, Davood Anvari, Seth Christopher Yaw Appiah, Muhammad Aqeel, Jalal Arabloo, Mohammad Asghari Jafarabadi, Marcel Ausloos, Atif Amin Baig, Maciej Banach, Till Winfried Bärnighausen, Krittika Bhattacharyya, Zulfiqar A. Bhutta, Ali Bijani, Oliver J. Brady, Nicola Luigi Bragazzi, Zahid A. Butt, Felix Carvalho, Vijay Kumar Chattu, Saad M. A. Dahlawi, Giovanni Damiani, Feleke Mekonnen Demeke, Kebede Deribe, Samath Dhamminda Dharmaratne, Daniel Diaz, Alireza Didarloo, Lucas Earl, Maysaa El Sayed Zaki, Maha El Tantawi, Nazir Fattahi, Eduarda Fernandes, Nataliya A. Foigt, Masoud Foroutan, Richard Charles Franklin, Yuming Guo, Arvin Haj-Mirzaian, Samer Hamidi, Hadi Hassankhani, Claudiu Herteliu, Tarig B. Higazi, Mostafa Hosseini, Mehdi Hosseinzadeh, Mowafa Househ, Olayinka Stephen Ilesanmi, Irena M. Ilic, Milena D. Ilic, Seyed Sina Naghibi Irvani, Ravi Prakash Jha, John S. Ji, Jost B. Jonas, Jacek Jerzy Jozwiak, Leila R. Kalankesh, Naser Kamyari, Behzad Karami Matin, Salah Eddin Karimi, Gbenga A. Kayode, Ali Kazemi Karyani, Ejaz Ahmad Khan, Md Nuruzzaman Khan, Khaled Khatab, Mona M. Khater, Neda Kianipour, Yun Jin Kim, Soewarta Kosen, Dian Kusuma, Carlo La Vecchia, Van Charles Lansingh, Paul H. Lee, Shanshan Li, Shokofeh Maleki, Mohammad Ali Mansournia, Francisco Rogerlândio Martins-Melo, Colm McAlinden, Walter Mendoza, Tomislav Mestrovic, Masoud Moghadaszadeh, Abdollah Mohammadian-Hafshejani, Seyyede Momeneh Mohammadi, Shafiu Mohammed, Rahmatollah Moradzadeh, Paula Moraga, Mehdi Naderi, Ahamarshan Jayaraman Nagarajan, Ionut Negoi, Cuong Tat Nguyen, Huong Lan Thi Nguyen, Bogdan Oancea, Andrew T. Olagunju, Ahmed Omar Bali, Obinna E. Onwujekwe, Adrian Pana, Vafa Rahimi-Movaghar, Kiana Ramezanzadeh, David Laith Rawaf, Salman Rawaf, Reza Rawassizadeh, Aziz Rezapour, Ana Isabel Ribeiro, Abdallah M. Samy, Masood Ali Shaikh, Kiomars Sharafi, Aziz Sheikh, Jasvinder A. Singh, Eirini Skiadaresi, Shahin Soltani, Wilma A. Stolk, Mu’awiyyah Babale Sufiyan, Alan J. Thomson, Bach Xuan Tran, Khanh Bao Tran, Bhaskaran Unnikrishnan, Francesco S. Violante, Giang Thu Vu, Tomohide Yamada, Sanni Yaya, Paul Yip, Naohiro Yonemoto, Chuanhua Yu, Yong Yu, Maryam Zamanian, Yunquan Zhang, Zhi-Jiang Zhang, Arash Ziapour, Simon I. Hay, Simon I. Hay

**Affiliations:** Institute for Health Metrics and Evaluation, University of Washington, Seattle, WA 98121 USA

**Keywords:** Onchocerciasis, Geospatial model, Neglected tropical diseases

## Abstract

**Background:**

Onchocerciasis is a disease caused by infection with *Onchocerca volvulus*, which is transmitted to humans via the bite of several species of black fly, and is responsible for permanent blindness or vision loss, as well as severe skin disease. Predominantly endemic in parts of Africa and Yemen, preventive chemotherapy with mass drug administration of ivermectin is the primary intervention recommended for the elimination of its transmission.

**Methods:**

A dataset of 18,116 geo-referenced prevalence survey datapoints was used to model annual 2000–2018 infection prevalence in Africa and Yemen. Using Bayesian model-based geostatistics, we generated spatially continuous estimates of all-age 2000–2018 onchocerciasis infection prevalence at the 5 × 5-km resolution as well as aggregations to the national level, along with corresponding estimates of the uncertainty in these predictions.

**Results:**

As of 2018, the prevalence of onchocerciasis infection continues to be concentrated across central and western Africa, with the highest mean estimates at the national level in Ghana (12.2%, 95% uncertainty interval [UI] 5.0–22.7). Mean estimates exceed 5% infection prevalence at the national level for Cameroon, Central African Republic, Democratic Republic of the Congo (DRC), Guinea-Bissau, Sierra Leone, and South Sudan.

**Conclusions:**

Our analysis suggests that onchocerciasis infection has declined over the last two decades throughout western and central Africa. Focal areas of Angola, Cameroon, the Democratic Republic of the Congo, Ethiopia, Ghana, Guinea, Mali, Nigeria, South Sudan, and Uganda continue to have mean microfiladermia prevalence estimates exceeding 25%. At and above this level, the continuation or initiation of mass drug administration with ivermectin is supported. If national programs aim to eliminate onchocerciasis infection, additional surveillance or supervision of areas of predicted high prevalence would be warranted to ensure sufficiently high coverage of program interventions.

**Supplementary Information:**

The online version contains supplementary material available at 10.1186/s12916-022-02486-y.

## Background

Onchocerciasis is a disease caused by infection with the filarial nematode *Onchocerca volvulus*, which is transmitted to humans by the bite of the infected black fly (*Simulium* spp.). Over time, infection can cause permanent blindness or severe skin manifestations, including extreme and debilitating itching. Formerly endemic in focal areas of the Americas, the global distribution of onchocerciasis is now entirely concentrated in Africa and Yemen [1]. Interventions to control or interrupt transmission have been implemented since the mid-1970s, either through vector control (larviciding) or, since the late 1980s, using mass drug administration (MDA) with ivermectin. Preventive chemotherapy with MDA (in which all eligible individuals residing in endemic areas are offered ivermectin) is currently the primary intervention for the control of morbidity and elimination of transmission, largely delivered via the Community-Directed Treatment with Ivermectin (CDTI) strategy [2]. Over 1 billion ivermectin treatments have been donated to national onchocerciasis control programs, in addition to millions of treatments provided under the auspices of national lymphatic filariasis (LF) elimination programs.

Evidence from settings in Uganda [3] and Sudan [4], from the Onchocerciasis Elimination Program in the Americas [5, 6], and from modeling studies [7, 8] suggests there is a possibility that annual or more frequent MDA reaching at least 80% of the eligible population may halt transmission. The success of local elimination has led national programs, donors, implementing partners, the Mectizan Donation Program, and technical experts to consider the feasibility of onchocerciasis elimination in Africa [9]. Elimination is achieved as transmission is first suppressed through > 80% population coverage with annual MDA, and then ultimately interrupted as the reservoir of prevalent adult worms experiences mortality or infertility [10]. The duration of MDA required to eliminate transmission in Africa will vary by individual setting, with projections from simulation studies ranging from 10 to 25 years, depending on baseline prevalence and intensity of infection, population MDA coverage, and other local factors. Operational research is currently underway to refine guidelines for evaluating elimination programs, improve diagnostic test performance, and develop new therapeutics. Deployment of novel intervention strategies such as “test-and-not-treat” [11] is also being evaluated in areas co-endemic for *O. volvulus* and the filarial nematode *Loa loa*. MDA with ivermectin is contraindicated among individuals with loiasis due to a significant potential for severe neurological outcomes. The risk of severe adverse events may outweigh the benefits of ivermectin MDA in areas that are both endemic for loiasis and hypoendemic for onchocerciasis. Spatial prediction of onchocerciasis burden could benefit control programs by helping identify locations where alternative strategies may be needed for safe and effective elimination [12].

Achieving elimination of onchocerciasis transmission in Africa will require investment across the continent, from mapping surveys to identify and confirm areas requiring MDA to periodic monitoring of program impact in human and vector populations over at least a decade following initiation of interventions. According to the Expanded Special Project for the Elimination of Neglected Tropical Diseases (ESPEN), nearly 2000 districts may require some form of data collection to confirm eligibility for MDA with ivermectin [13]. Since local factors such as vector subspecies, human movement, and environmental conditions contribute to local variation in onchocerciasis prevalence, model-based geostatistics offers an opportunity to integrate the spatial and temporal relations in the existing evidence base to predict prevalence of onchocerciasis infection continuously, augmented with covariates to capture variation in the distribution of infection at finer spatial scales (see for example Cromwell et al. 2020) [14]. While these predictions are no substitute for primary data collection, they can be used to guide prioritization of areas to survey or targeted strengthening of MDA interventions. Such models have been used previously to estimate the pre-control [15] prevalence of skin snip positivity for the west African context as well as nodule prevalence for areas supported by the African Programme for Onchocerciasis Control [16]. To date, there are no contemporary geospatial estimates for the entire African continent or Yemen.

The objective of this analysis was to estimate the prevalence of onchocerciasis infection across the African continent and Yemen through time, quantifying the progress achieved in reducing onchocerciasis infection from 2000 to 2018, by accounting for ivermectin MDA implemented by national onchocerciasis control programs, as well as for the purpose of eliminating lymphatic filariasis as a public health problem. We also stratify these estimates of the number infected among areas identified to be high-risk for *L. loa*, as novel implementation strategies such as “test-and-not-treat” [11] will be required to achieve onchocerciasis elimination in these locations.

## Methods

### Data inputs

Data on the prevalence of onchocerciasis infection is largely collected by national onchocerciasis control and elimination programs as part of routine program monitoring. While methods for data collection vary by time and place, areas covered by the former Onchocerciasis Control Programme (OCP) in west Africa and the African Programme for Onchocerciasis Control (APOC), as well as onchocerciasis control programs supported by other partners, often identified areas (foci or districts) eligible for MDA or vector control by purposively sampling communities near known or suspected *Simulium* breeding sites. In OCP-supported areas, prevalence of onchocerciasis was estimated using skin snip biopsy (microscopy) to detect the presence of microfilariae; in APOC-areas, nodule (onchocercoma) palpation was used in the rapid epidemiological mapping for onchocerciasis (REMO) [17]. More recently, onchocerciasis programs have used Ov16 antibody testing by ELISA (enzyme-linked immunosorbent assay), in conjunction with entomological surveillance, as per WHO guidelines [10] to demonstrate elimination of transmission, and the use of rapid diagnostic tests is being evaluated for programmatic use. We compiled an analytical dataset of onchocerciasis infection prevalence from the following sources: a systematic review of literature in which data collected between 1988 and the present were included in the analysis (Additional file 1: Fig. S2 and Table S3); the ESPEN online portal [18]; and personal communication for data collected under the OCP [15] from its former Director, BA Boatin, PhD (personal communication, January 2019). Data were reviewed and geo-referenced either to point locations (i.e., a community) or polygons (i.e., areal data attributed to a focus or district). In this analysis, we included data for which nodule palpation or skin snip biopsy was reported. A total of 17,896 point-referenced and 220 polygon-referenced inputs were included in the analysis, with 14,314 total inputs initially reported as nodule prevalence and 3,802 as skin snip biopsy. Further details on the dataset are presented in Additional file 1: Section 3.

### Geospatial covariates

In order to develop a predictive model of onchocerciasis prevalence that was generalizable to under-surveyed locations and years, we sought to include a suite of environmental covariates that may be associated with the presence or intensity of *O. volvulus* transmission (Additional file 1: Table S5 and Fig. S4). We compiled covariates that collectively provide a broad characterization of local ecological conditions, including precipitation, temperature, aridity, orographic slope, vegetation, soil characteristics, distance to rivers, and maximum river width. Human population density was also included to accommodate a possible association with urbanicity. Cumulative years of any MDA with ivermectin for onchocerciasis or lymphatic filariasis (as a single covariate) were included. Finally, we included outputs from a recent model of onchocerciasis environmental suitability (Additional file 1: Fig. S8; Cromwell and colleagues [19]) to incorporate environmental effects calibrated by onchocerciasis presence data. Raw covariate raster surfaces were resampled to a consistent 5 × 5-km grid-cell resolution (see Additional file 1: Section 4.1). Time-varying covariates (e.g., climatic variables and interventions) were associated with their corresponding model years, except when specific years of data were unavailable for a given covariate, in which case the nearest available year of data was used (covariate temporal coverage is listed in Additional file 1: Table S5). Analysis of variance inflation factors [20] (VIF, with a VIF threshold of 3.0) was used to exclude collinear covariates (Additional file 1: Section 4.3). Model reliability is affected by the overlap between covariate values in training and prediction datasets (see Additional file 1: Fig. S7). Predictions in regions with covariate values falling outside the range of training values may be prone to extrapolation errors and should be considered with special caution. Such areas include the Sahel and Sahara, Yemen, Kenya, Somalia, eastern Ethiopia, and southern Angola.

### Age and diagnostic adjustment

In order to derive global estimates of onchocerciasis infection using data reported across different age ranges and diagnostic tests, we used age and diagnostic models to adjust (“crosswalk”) input data prior to the main modeling analysis, yielding estimates of both-sex, all-age (0–94 years) microfiladermia prevalence as measured by skin snip microscopy. To develop models to adjust age-specific data to all-age prevalence or to adjust nodule prevalence data to skin snip microscopy, we identified peer-reviewed published surveys that reported skin snip or nodule prevalence, or both, in multiple age groups within the same study populations, from countries included in the geospatial modeling region (Additional file 1: Table S6). Diagnostic effects and non-linear prevalence-by-age relationships were estimated simultaneously by maximum likelihood optimization of a logistic regression model, using separate basis splines on age for each diagnostic test (skin snips and nodules), an indicator variable for skin snip surveys, and study population-level fixed effects. Scaling factors were then estimated for each observation in the full geospatial modeling dataset, by fixing model coefficients to the mean estimates derived from the training set and optimizing the study population-level effects via maximum likelihood. Reported prevalence values were adjusted by applying these scaling factors to the inferred (age and diagnostic models) all-age prevalence curves for the reported diagnostic type, yielding estimates of all-age skin snip prevalence. These crosswalked prevalence values were used as outcome data in the geospatial model. Further details about the diagnostic and age adjustment methodology and results are provided in Additional file 1: Section 5.1.

### Geostatistical analysis

A Bayesian geostatistical model [21, 22] was fit for the group of African countries (plus Yemen) known or suspected to include locations endemic for onchocerciasis as defined by ESPEN. Justification of the geographical restrictions used to establish the modeling region is presented in Additional file 1: Section 3 and Table S2. While we were primarily concerned with prevalence estimates for the time period 2000–2018, we fit the model using data from 1988 to 2018 in order to incorporate data from pre-2000 OCP and APOC surveys and thereby improve estimates in countries covered by those programs. Reporting of results focuses on estimates for 2000–2018.

The full onchocerciasis prevalence model was a spatial generalized linear mixed effects model using a binomial likelihood and minimally informative priors (Additional file 1: Section 5.3 and Table S7). The model was estimated by integrated nested Laplace approximation (INLA) [23] within the R package R-INLA [24]. Covariates were included as fixed effects, except that estimates from the onchocerciasis suitability model were incorporated using a second-order random walk model to accommodate non-linearity. The model included country-level random effects to account for variation in national onchocerciasis burdens and control programs, and a nugget variance term to accommodate fine-spatial scale and sampling variation. A spatial Gaussian process was used to model residual spatial variation, using stochastic partial differential equations (SPDE) [25] and a Matérn spatial covariance function. Predictions were generated at a 5 × 5-km spatial resolution, with 1,000 samples drawn from the joint posterior distribution. Predictions were summarized using the means and 95% uncertainty intervals (UI; 2·5th and 97·5th percentiles) from the 1,000 draws of prevalence.

Aggregate estimates of onchocerciasis prevalence were calculated using population-weighted means of grid-cell-level prevalence, with weighting by WorldPop [26] grid-cell-level modeled population estimates calibrated to match Global Burden of Disease population estimates at national or administrative subunit level 1 (where available). Estimates were aggregated across 5 × 5-km cells within administrative boundaries at national and administrative levels 1 and 2, using updated administrative shapefiles originally supplied by GADM (Global Administrative Areas) [27]. We first masked all final model outputs for which land cover was classified as “barren or sparsely vegetated” by Moderate Resolution Imaging Spectroradiometer satellite data for 2015 [28], as well as areas in which total population density in 2015 was less than ten individuals per 1 × 1-km grid cell by WorldPop population estimates. Estimates from such locations (e.g., the southern Sahara Desert) are considered less reliable due to sparse prevalence data sampling and extreme covariate values. We retained input data from such areas in the model because they are still informative about the spatial distribution of onchocerciasis prevalence and its relationship with model covariates.

Five-fold cross-validation was used for out-of-sample model validation. The geostatistical model was run five times, each time holding out data from one spatially stratified fold and generating predictions for the held-out data. A suite of measures of out-of-sample performance were examined, namely bias, mean absolute error, root mean square error, 95% prediction interval data coverage, and correlations of observed to predicted values. The data processing and modeling workflows for this study are outlined in Additional file 1: Fig. S1. All statistical analysis was performed using statistical software R v.3.5.1.

## Results

As of 2018, the prevalence of onchocerciasis infection continues to be concentrated across central Africa, with the highest prevalence areas in focal areas of the Democratic Republic of the Congo (DRC), Ghana, Nigeria, Cameroon, and South Sudan, based on mean predictions at the 5 × 5-km resolution. Mean prevalence predictions were also above 10% in focal areas of several additional countries, including Angola, Ethiopia, Gabon, Nigeria, and the Republic of the Congo. Mean prevalence at the national level was highest in Ghana (12.2%, 95% uncertainty interval [UI] 5.0–22.7) and Equatorial Guinea (9.7%, 8.0–11.7), with mean estimates also exceeding 5% infection prevalence at the national level for Cameroon, Central African Republic, DRC, Guinea-Bissau, Sierra Leone, and South Sudan.

Our model estimates should be considered in the context of model performance (Additional file 1: Fig. S11 and Table S9). Overall out-of-sample bias was low, with a mean error of 0.003 (0.3% in prevalence space) across all model years (1988–2018). The variation over time and space of mean error and other performance metrics, including mean absolute error (overall value: 0.111, or 11.1%), RMSE (overall value: 0.168, or 16.8%), and correlation (overall value: 0.706), and the sometimes wide uncertainty intervals of predictions (both in- and out-of-sample) reflect in part limited data on onchocerciasis infection prevalence across the time series for many locations. In other areas where data are unavailable, such as southern Kenya or the border between Sudan and South Sudan, covariate patterns are under-represented in the input data and our predictions should be interpreted in conjunction with other programmatic data sources.

**Fig. 1:**
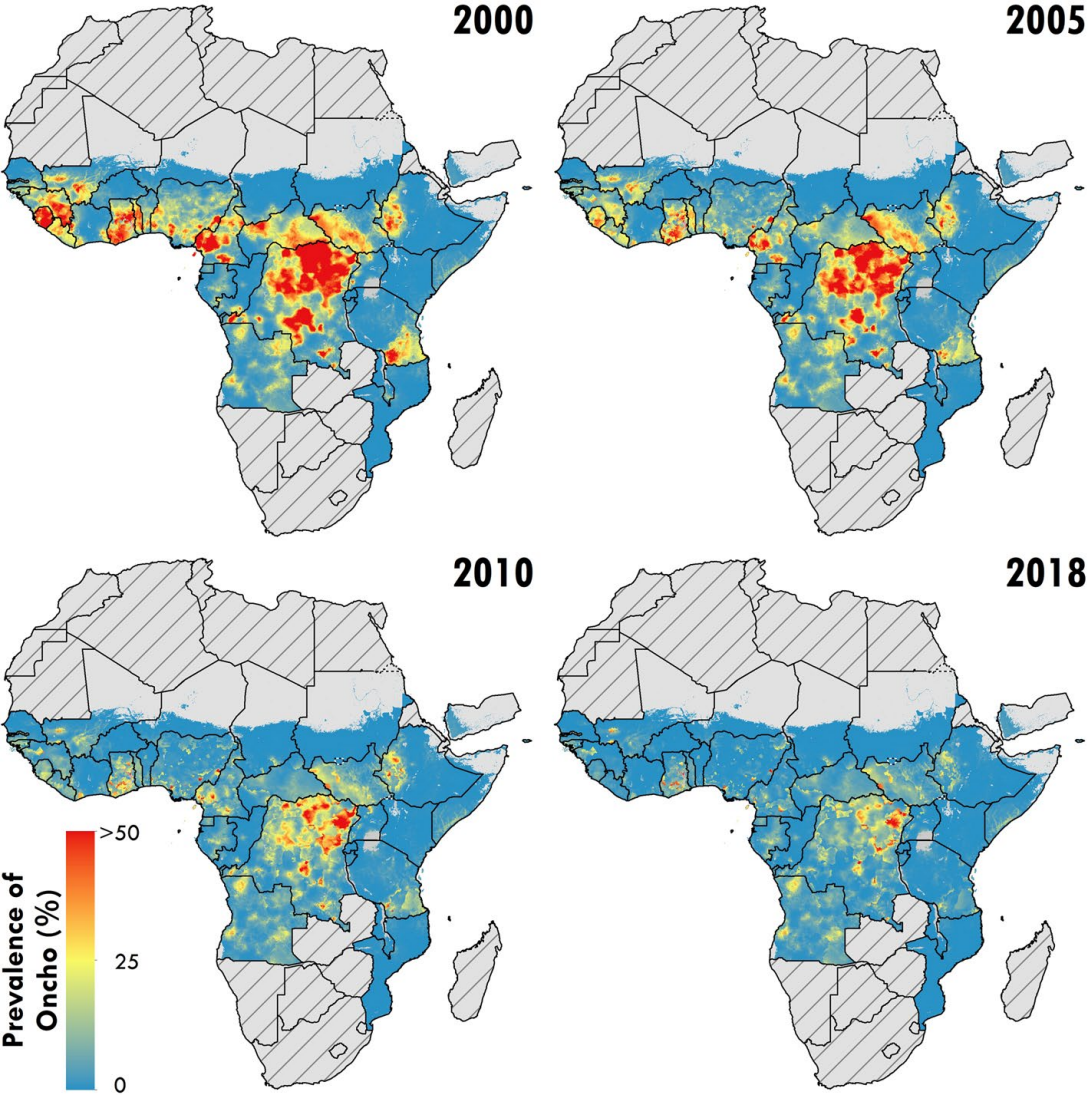
Prevalence of *O. volvulus* microfiladermia in Africa and Yemen at the 5 × 5-km level. Mean predictions of *O. volvulus* microfiladermia prevalence (all-age, both sexes) from the Bayesian geostatistical model, as measured by skin snip biopsies and crosswalked nodule palpation surveys. Hatch-marks indicate countries for which estimates were not produced; grey areas are masked based on sparsely populated areas (fewer than ten people per 1 × 1-km grid cell) and barren landscape classification. Data can be viewed on an interactive visualization tool at https://vizhub.healthdata.org/lbd/oncho

**Fig. 2:**
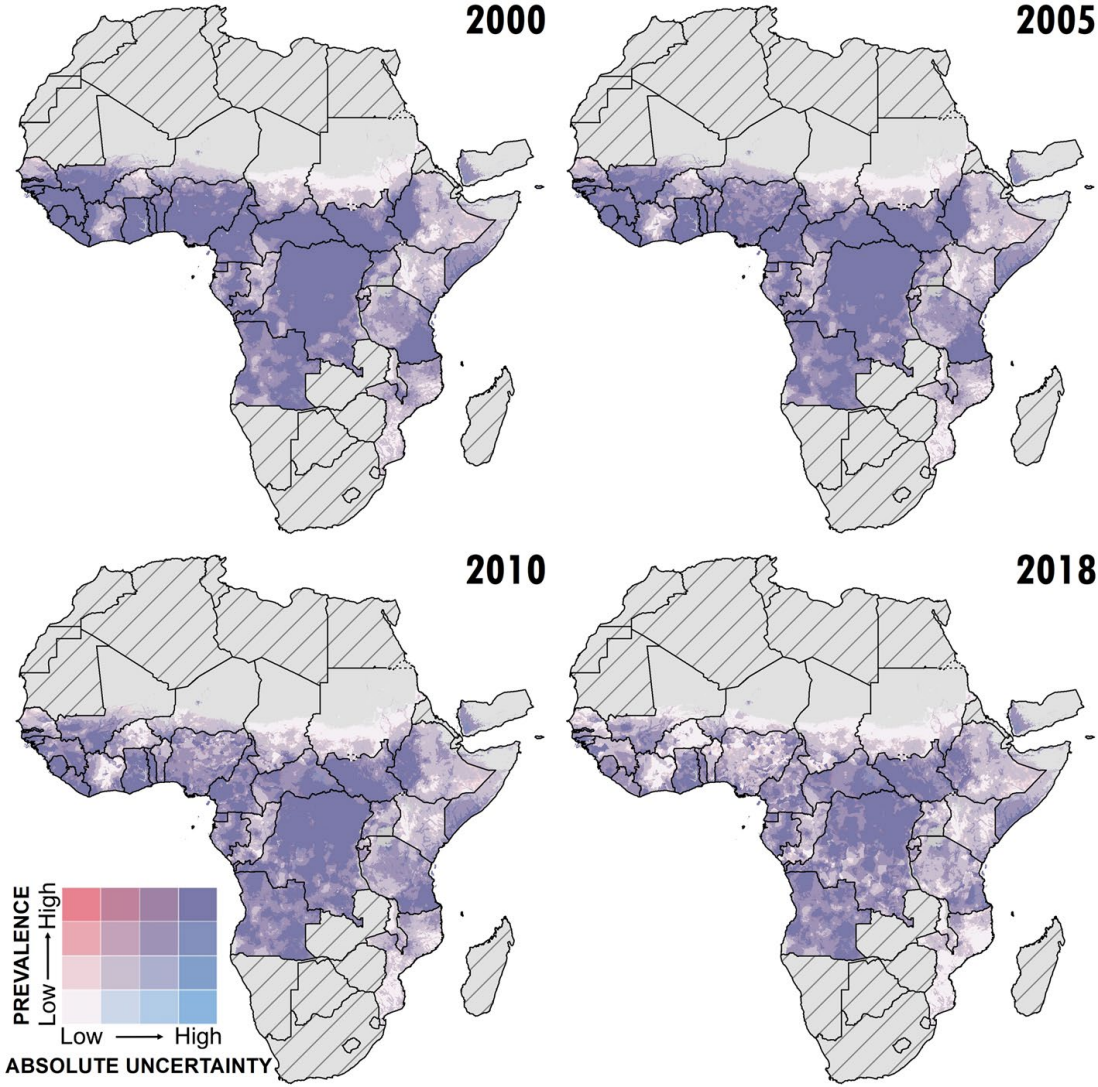
Africa and Yemen *O. volvulus* microfiladermia model uncertainty at the 5 x 5-km level. Mean and absolute uncertainty (measured as the range, or difference between, the upper and lower 95% UI) in *O. volvulus* microfiladermia prevalence estimates (all-age, both sexes) in Africa and Yemen. Hatch-marks indicate countries for which estimates were not produced; grey areas are masked based on sparsely populated areas (fewer than ten people per 1 × 1-km grid cell) and barren landscape classification. Quantile breakpoints for plotted categories are 0.001 (25th percentile), 0.009 (50th percentile), and 0.048 (75th percentile) for mean prevalence, and 0.009, 0.054, and 0.258 for range. Data can be viewed on an interactive visualization tool at https://vizhub.healthdata.org/lbd/oncho.

As illustrated in Fig. 1, while the analysis shows large declines overall in the prevalence of onchocerciasis from 2000 to 2018, much of central Africa would continue to warrant MDA with ivermectin (among districts for which *Loa loa* is non-endemic) or consideration for “test-and-not-treat” implementation in areas where MDA might be broadly contraindicated due to high loiasis burden. In central Africa, much of the high infection prevalence is, in part, among areas ineligible for ivermectin due to loiasis burden, or areas of greater insecurity or inaccessibility. The model predicts low (under 1%) infection prevalence for nearly all areas in northern and central Burkina Faso, central and eastern Niger, northern Guinea, northern Côte d’Ivoire, eastern Ethiopia, Kenya, and much of Tanzania. The uncertainty (Fig. 2) of these predictions is high, particularly for estimates from 2000 to 2005, at both the fine-spatial scale (5 × 5-km resolution), as well as national and subnational-level predictions. Detailed model results, including uncertainty results and temporal trends, are also available for scrutiny in an interactive visualization tool at https://vizhub.healthdata.org/lbd/oncho.

**Fig. 3:**
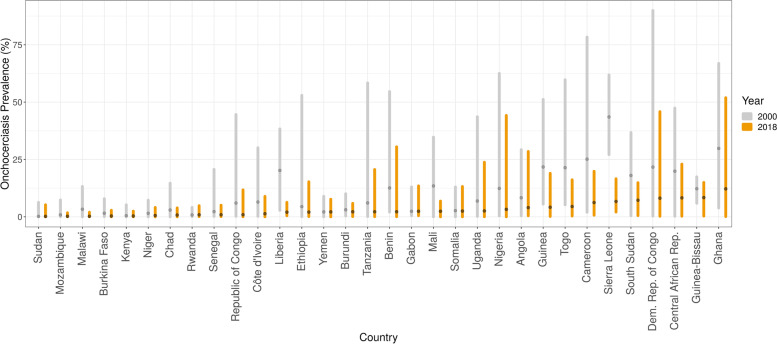
Distribution of onchocerciasis prevalence within and among countries at administrative level 2 (2000 and 2018). The median (central points) and lowest–highest (bars) mean prevalence estimates at administrative level 2 are shown for each country in the modeling region, for 2000 and 2018. Countries are ordered by increasing median administrative level 2 prevalence in 2018

For the period 2000–2018, most national onchocerciasis programs aimed to control morbidity, not eliminate transmission. As such, high-burden areas typically received interventions with CDTi. In Fig. 3, we present the median, minimum, and maximum second-order administrative unit-level prevalence estimates for 2000 and 2018. This comparison illustrates the reductions in infection prevalence achieved, narrowing the gap between high-burden and low-burden districts. Such reductions are most notable in Cameroon, Ghana, and Sierra Leone. In areas of Uganda and Sudan known to have achieved elimination of transmission, our model predictions are consistent with observed data. In the Abu Hamed focus (Sudan), our results are consistent with elimination targets being met by 2007 [29]. In Uganda, our model results are consistent with program progress in the 15 foci for which MDA has ceased [30].

## Discussion

This analysis quantifies the impact of nearly two decades of national onchocerciasis control and elimination program activities. While there have been substantial reductions in the overall prevalence of infection that should be celebrated, like so many health metrics [22, 31], this has not been achieved equally throughout the continent. Prior geospatial analyses of onchocerciasis prevalence [15, 16] have been limited to regional estimates for west Africa and the APOC areas (central and eastern Africa). Our analysis is qualitatively very similar to these previous estimates of high nodule prevalence, showing particularly high burden in the DRC [16]. This is expected, as the relationship between nodule prevalence and skin snip positivity has better agreement at higher levels of prevalence [32]. Although our model includes data collected throughout the continent for both skin snip and nodule prevalence (with nodule prevalence adjusted to represent prevalence of infection measured via skin snip), our model predictions for 2011 (available via the online visualization) are qualitatively similar to the estimates published by Zouré and colleagues [16]. Our analysis extends prediction to include areas masked from that analysis based on expert opinion on suitability, as we intend these model results to be useful for decision-making by national programs for locations under consideration for onchocerciasis elimination mapping, in conjunction with the best local evidence.

We envision three specific use cases for this analysis. First, overall onchocerciasis burden estimates should reflect the implementation of nearly 20 years of MDA for both onchocerciasis and lymphatic filariasis programs. This geospatial model will be used in future updates to the Global Burden of Disease Study [33] to adjust estimates of contemporary morbidity due to onchocerciasis. Second, as national onchocerciasis programs are currently in the process of consolidating historical evidence, plans for elimination of transmission including additional mapping surveys and MDA will require such evidence to be aggregated for decision-making and prioritization. In light of limited resources, programs may wish to consider using these maps, alongside other data sources, to identify priority areas or evaluate potential low-endemicity areas for program eligibility. In the case of areas potentially co-endemic for LF and onchocerciasis, these results may enable program managers to prioritize timing of post-MDA surveillance for both pathogens, rather than ceasing MDA for LF while onchocerciasis infection may still be prevalent. Third, in areas co-endemic for *Loa loa*, this analysis may serve to quantify program targets for novel MDA implementation strategies.

By producing estimates for all known endemic countries in Africa and Yemen, this analysis is comprehensive in scope for the locations currently under consideration for the elimination of transmission. We conducted a systematic literature review to identify historical prevalence data and included publicly available prevalence data provided by national programs via the ESPEN online data portal. Additional data from the former OCP areas were also included, substantially strengthening our predictions for west Africa. We also developed models for age and diagnostic adjustment in order to leverage data reported across multiple age categories and reconcile the two dominant diagnostic methods employed in program monitoring. Our approach using model-based geostatistics enables us to predict prevalence while accounting for a broad range of covariates associated with onchocerciasis and other neglected tropical diseases optimized for prediction, to be of maximal utility to programs. We developed a geospatial covariate of MDA with ivermectin to account for the impact of both onchocerciasis and lymphatic filariasis programs.

The analysis has several limitations we wish to acknowledge. First, it is possible that covariate patterns do not adequately capture the ecological niche for *Simulium* in all settings, particularly given the flight range of the vector exceeds 5 km [34]. Combined with human movement, it is possible that the locations for which communities test positive may not directly correlate with where individuals are infected. *Simulium* density data are not widely available; therefore, we are unable to include measures of the vector as a covariate. These model results could be compared against more detailed remote sensing analyses for specific locations; however, the fine spatial scale of those approaches would be computationally infeasible at the continental scale. Further, we do not have complete enumeration of breeding sites, and so the analysis assumes other covariates represent ecological conditions that might be suitable for transmission and are a sufficient proxy for exposure to both the vector and *O. volvulus*. There may be settings where seasonal rivers enable establishment of viable breeding sites, and future analysis could consider more detailed hydrological data sources. We excluded serological prevalence data inputs, as the relationship between antibody positivity and population-level infection prevalence was unstable, and variability exists in the performance of specific antibody-based diagnostic methods or protocols [35]. Less than 1% of the total input data we obtained was measured using serological tests (from a total of seven countries), and exclusion of these data from preliminary models resulted in negligible differences in the results. Prevalence of microfilariae measured by skin snip biopsy is also subject to limitations as sensitivity is lower in low-prevalence settings. Future work should consider the possibility of false negatives, particularly in pre-control data inputs. Future work is needed to incorporate Ov16 serological tests into the modeling framework, as more programs will use this diagnostic for end of program surveillance, as well as baseline mapping of districts for which contemporary evidence is unavailable. While our model does include MDA as a covariate, we did not use reported coverage (i.e., percentage of the population that received treatment). Data on reported coverage by district are unavailable for all implementation units across the time series, and reported coverage has been demonstrated to be biased [36]. We did not include explicit temporal terms in the model because extensive time series exist for relatively few locations (see the spatial and temporal distribution of available data in Additional file 1: Fig. S3), and exploratory spatiotemporal models yielded unrealistically erratic temporal trends. Allowing temporal changes in prevalence to be driven by the covariates produced more tenable trends, but the resulting model may be insensitive to particularly rapid prevalence changes in some localities. Finally, as prevalence data were collected for the purposes of program monitoring, there is likely heterogeneity in the quality of field-based data collection that we are unable to account for in this model.

## Conclusions

The feasibility of elimination of onchocerciasis transmission throughout Africa is currently under consideration by national programs, implementing partners, donors, and drug-donating pharmaceutical companies. While areas of high prevalence remain, our analysis shows that programs have been extremely successful in reducing prevalence across high-endemicity locations. We present the first time series estimates of infection prevalence to quantify the gains currently achieved by control and elimination interventions to assist with prioritization and program planning. It is for decision-makers at all levels to decide if elimination is a feasible goal.

## Supplementary Information

**Additional file 1: Supplementary Appendix.** This file contains a GATHER compliance checklist, a list of data sources used in the present analysis, and further details of the analytical methodology and results.

## Data Availability

Detailed model results, including uncertainty results, are available through an interactive visualization tool at https://vizhub.healthdata.org/lbd/oncho. The data sources and code used to generate these estimates, as well as tables of mean estimates and uncertainty intervals, are publicly available online at the Global Health Data Exchange (GHDx; http://ghdx.healthdata.org/).

## Abbreviations

APOC: African Programme for Onchocerciasis Control
CDTI: Community-Directed Treatment with Ivermectin
DRC: Democratic Republic of the Congo
ELISA: Enzyme-linked immunosorbent assay
ESPEN: Expanded Special Project for the Elimination of Neglected Tropical Diseases
GADM: Global Administrative Areas
INLA: Integrated nested Laplace approximation
LF: Lymphatic filariasis
MDA: Mass drug administration
OCP: Onchocerciasis Control Programme
REMO: Rapid epidemiological mapping for onchocerciasis
SPDE: Stochastic partial differential equations
VIF: Variance Inflation Factor
