# Supplementary material for: The prevalence of onchocerciasis in Africa and Yemen, 2000–2018: a geospatial analysis
Source: BMC Med. 2022 Sep 7;20:293. doi: 10.1186/s12916-022-02486-y (PMC9449300; doi:10.1186/s12916-022-02486-y)
Supplement: Supplementary file 1 — Additional file 1: Supplementary Appendix. This file contains a GATHER compliance checklist, a list of data sources used in the present analysis, and further details of the analytical methodology and results. [file 12916_2022_2486_MOESM1_ESM.docx]

**Additional File 1: Supplementary Appendix for *The prevalence of onchocerciasis in Africa and Yemen, 2000–2018: A geospatial analysis***

Contents

**[2.0 Supplementary discussion](#_Toc108451478)** [4](#_Toc108451478)

**[3.0 Supplementary data](#_Toc108451479)** [4](#_Toc108451479)

**[3.1 Geographical restrictions](#_Toc108451480)** [4](#_Toc108451480)

**[3.2 Systematic review](#_Toc108451481)** [7](#_Toc108451481)

[3.2.1 Systematic review data processing 7](#_Toc108451482)

**[3.3 Geo-positioning](#_Toc108451483)** [8](#_Toc108451483)

**[3.4 Data processing](#_Toc108451484)** [8](#_Toc108451484)

**[3.5 APOC survey year matching](#_Toc108451485)** [18](#_Toc108451485)

**[4.0 Supplementary covariates](#_Toc108451486)** [19](#_Toc108451486)

**[4.1 Pre-existing covariates considered for analysis](#_Toc108451487)** [19](#_Toc108451487)

**[4.2 Creation of MDA covariate](#_Toc108451488)** [19](#_Toc108451488)

**[4.3 Covariate reduction](#_Toc108451489)** [19](#_Toc108451489)

**[5.0 Supplementary methods](#_Toc108451490)** [25](#_Toc108451490)

**[5.1 Age and diagnostic crosswalks](#_Toc108451491)** [25](#_Toc108451491)

**[5.2 Polygon resampling](#_Toc108451492)** [30](#_Toc108451492)

**[5.3 Geostatistical model](#_Toc108451493)** [32](#_Toc108451493)

[5.3.1 Model geographies and time period 32](#_Toc108451494)

[5.3.2 Covariate coverage 32](#_Toc108451495)

[5.3.3 Environmental suitability 32](#_Toc108451496)

[5.3.4 Model description 34](#_Toc108451497)

[5.3.5 Priors 35](#_Toc108451498)

[5.3.6 Mesh construction 35](#_Toc108451499)

[5.3.7 Model fitting and estimation generation 36](#_Toc108451500)

[5.3.8 Model results 36](#_Toc108451501)

[5.3.9](#_Toc108451502) *[Loa loa](#_Toc108451502)* [endemicity 38](#_Toc108451502)

**[5.4 Model validation](#_Toc108451503)** [38](#_Toc108451503)

[5.4.1 Metrics of predictive validity 38](#_Toc108451504)

**[6.0 Supplementary references](#_Toc108451505)** [42](#_Toc108451505)

**List of Supplementary Figures**

[Supplementary Figure 1. Flowchart of major steps in data processing and modelling of onchocerciasis prevalence. 4](#_Toc98166857)

[Supplementary Figure 2. Onchocerciasis article review and data extraction flowchart. 8](#_Toc98166858)

[Supplementary Figure 3. Africa and Yemen data coverage maps. 17](#_Toc98166859)

[Supplementary Figure 4. Africa and Yemen covariate values. 25](#_Toc98166860)

[Supplementary Figure 5. Diagnostic and age crosswalk model. 29](#_Toc98166861)

[Supplementary Figure 6. Polygon resampling. 31](#_Toc98166862)

[Supplementary Figure 7. Number of covariates with values outside the central 95% interval of values at survey sites. 33](#_Toc98166863)

[Supplementary Figure 8. Onchocerciasis suitability model predictions (Cromwell et al., in review) for 2016. 34](#_Toc98166864)

[Supplementary Figure 9. Spatial mesh construction. 36](#_Toc98166865)

[Supplementary Figure 10. Estimated random effects for environmental suitability. 38](#_Toc98166866)

[Supplementary Figure 11. Model validation scatterplots for Africa and Yemen. 40](#_Toc98166867)

**List of Supplementary Tables**

[1.0 GATHER compliance 3](#_Toc98166868)

[Supplementary Table 1. Guidelines for Accurate and Transparent Health Estimates Reporting (GATHER) checklist. 3](#_Toc98166869)

[Supplementary Table 2. Geographical restrictions. 5](#_Toc98166870)

[Supplementary Table 3. Citations for data inputs. 9](#_Toc98166871)

[Supplementary Table 4. APOC REMO survey year investigations. 18](#_Toc98166872)

[Supplementary Table 5. Covariates considered or retained for modelling, 1988–2018. 21](#_Toc98166873)

[Supplementary Table 6. Data used in estimation of age and diagnostic crosswalk. 26](#_Toc98166874)

[Supplementary Table 7. INLA model priors. 35](#_Toc98166875)

[Supplementary Table 8. Parameter estimates from in-sample onchocerciasis MBG model. 37](#_Toc98166876)

[Supplementary Table 9. Out-of-sample validation metrics at the level of individual datapoints, from five-fold cross-validation 41](#_Toc98166877)

**1.0 GATHER compliance**

**Supplementary Table 1. Guidelines for Accurate and Transparent Health Estimates Reporting (GATHER) checklist.**

| **Item #** | **Checklist item** | **Reported on page #** |
| --- | --- | --- |
| **Objectives and Funding** | | |
| **1** | Define the indicator(s), populations (including age, sex, and geographic entities), and time period(s) for which estimates were made. | Main Text: Introduction, Methods (Data inputs); Methods (Covariates) |
| **2** | List the funding sources for the work. | Main text: Acknowledgments |
| **Data Inputs** | | |
| *For all data inputs from multiple sources that are synthesized as part of the study:* | | |
| **3** | Describe how the data were identified and how the data were accessed. | Main Text: Methods (Data inputs); Supplementary Information: 3.2 Systematic review |
| **4** | Specify the inclusion and exclusion criteria. Identify all ad-hoc exclusions. | Main Text: Methods (Data inputs); 3.0 Supplementary data; Supplementary Information: 3.2 Systematic review |
| **5** | Provide information on all included data sources and their main characteristics. For each data source used, report reference information or contact name/institution, population represented, data collection method, year(s) of data collection, sex and age range, diagnostic criteria or measurement method, and sample size, as relevant. | Supplementary Information: 3.0 Supplementary data |
| **6** | Identify and describe any categories of input data that have potentially important biases (e.g., based on characteristics listed in item 5). | Main text: Discussion (Limitations) |
| *For data inputs that contribute to the analysis but were not synthesized as part of the study:* | | |
| **7** | Describe and give sources for any other data inputs. | Main text: Methods (Geospatial covariates), Supplementary Information: 4.0 Supplementary covariates |
| *For all data inputs:* | | |
| **8** | Provide all data inputs in a file format from which data can be efficiently extracted (e.g., a spreadsheet rather than a PDF), including all relevant meta-data listed in item 5. For any data inputs that cannot be shared because of ethical or legal reasons, such as third-party ownership, provide a contact name or the name of the institution that retains the right to the data. | Available at (GHDx link will be added upon review)  Supplementary Information: 3.0 Supplementary data |
| **9** | Provide a conceptual overview of the data analysis method. A diagram may be helpful. | Main text: Methods (Age and diagnostic adjustment), Supplementary Information: 5.1 Age and diagnostic crosswalks; Supplementary Information: Table 6: data used in estimation of age and diagnostic crosswalk |
| **10** | Provide a detailed description of all steps of the analysis, including mathematical formulae. This description should cover, as relevant, data cleaning, data pre-processing, data adjustments and weighting of data sources, and mathematical or statistical model(s). | Main text: Methods (Geospatial analysis), Supplementary Information: 5.3 Geostatistical model |
| **11** | Describe how candidate models were evaluated and how the final model(s) were selected. | Main text: Methods (Geospatial analysis), Supplementary Information: 5.4 Model validation |
| **12** | Provide the results of an evaluation of model performance, if done, as well as the results of any relevant sensitivity analysis. | Main text: Methods (Geospatial analysis), Supplementary Information: 5.4 Model validation |
| **13** | Describe methods for calculating uncertainty of the estimates. State which sources of uncertainty were, and were not, accounted for in the uncertainty analysis. | Main text: Methods (Geospatial analysis), Supplementary Information: 5.3.4. Model description |
| **14** | State how analytic or statistical source code used to generate estimates can be accessed. | Available at (GHDx link will be added upon review) |
| **Results and Discussion** | | |
| **15** | Provide published estimates in a file format from which data can be efficiently extracted. | Raster files for spatial data and CSVs of estimates available at (GHDx link will be added upon review) |
| **16** | Report a quantitative measure of the uncertainty of the estimates (e.g., uncertainty intervals). | Supplementary Information: Supplementary Figure 11 and Table 9 |
| **17** | Interpret results in light of existing evidence. If updating a previous set of estimates, describe the reasons for changes in estimates. | Main text: Discussion (Strengths) |
| **18** | Discuss limitations of the estimates. Include a discussion of any modelling assumptions or data limitations that affect interpretation of the estimates. | Main text: Discussion  (Limitations) |

# **2.0 Supplementary discussion**

This document outlines the major data processing, modelling, and validation steps for the onchocerciasis prevalence analysis described in the main text (Supplementary Figure 1). We present a detailed description of model inputs, including data coverage, covariate sources, and geo-referencing. The geospatial model is described along with model validation metrics.


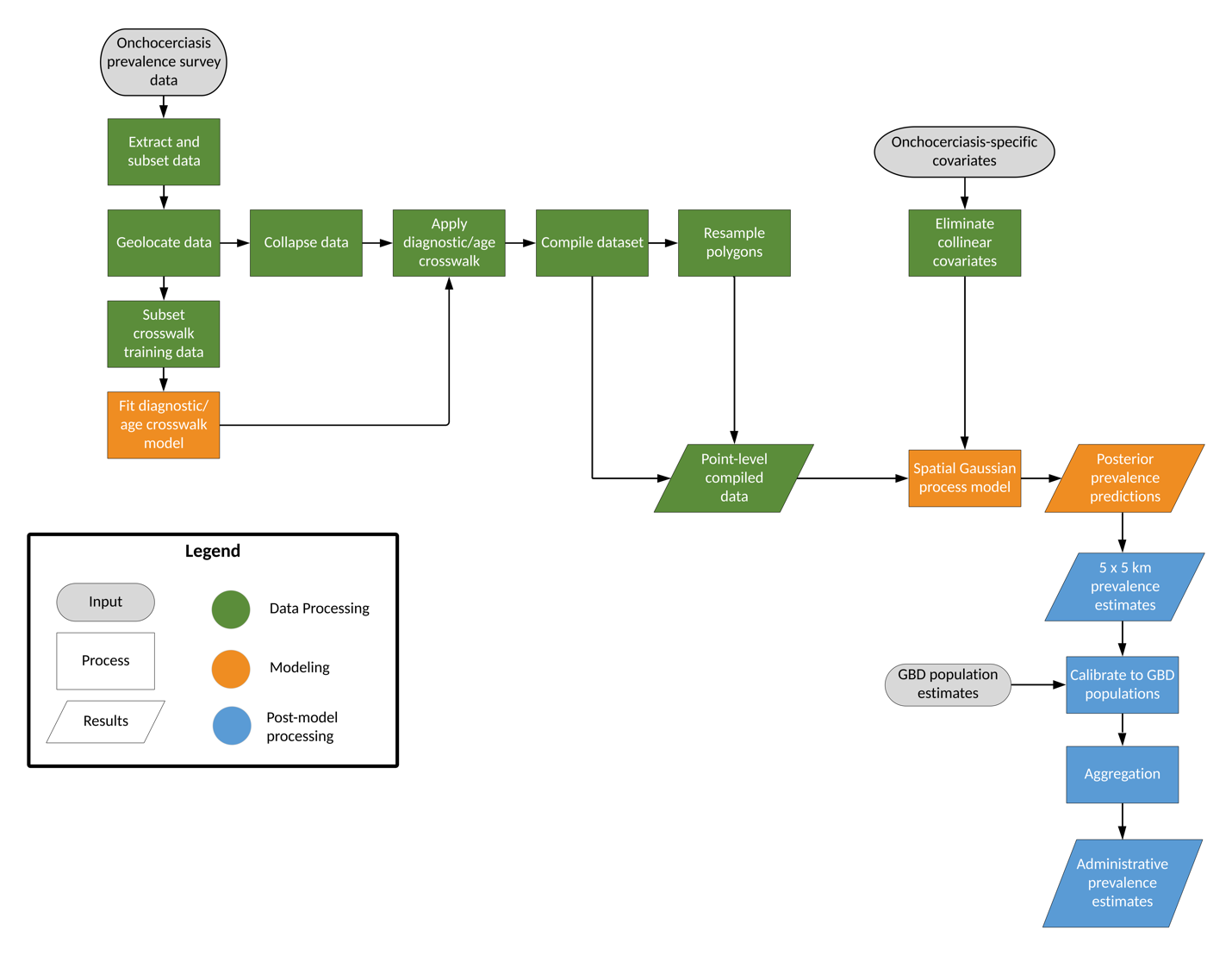


Supplementary Figure 1. Flowchart of major steps in data processing and modelling of onchocerciasis prevalence.

# **3.0 Supplementary data**

In the following section, we present a detailed summary of the data inputs used to estimate the prevalence of onchocerciasis in Africa and Yemen. Broadly, we aimed to include all published sources of onchocerciasis infection prevalence, as well as routine programme monitoring data collected to monitor progress toward onchocerciasis elimination. Data inputs were retained for analysis if they could be accurately geo-referenced.

## **3.1 Geographical restrictions**

Supplementary Table 2 lists all countries included in our MBG (model-based geostatistical) modelling region. Inclusion was partially based on ESPEN (Expanded Special Project for Elimination of Neglected Tropical Diseases) onchocerciasis endemicity classifications,(1) with extensions to some neighbouring countries outside the WHO (World Health Organization) AFRO (Regional Office for Africa) region which have evidence of onchocerciasis endemicity or uncertain endemicity status (Sudan, Somalia, and Yemen). Portions of Mauritius, Namibia, and Zambia are considered by ESPEN as candidates for future elimination mapping but were not included in our geospatial model. Conversely, The Gambia is considered non-endemic but we included it in our model for geographical continuity. Countries in the Americas with historical or residual onchocerciasis burdens were not modelled due to their highly localised endemicity.

**Supplementary Table 2. Geographical restrictions.**

The geographical definition of the modelling region is indicated, with country-level onchocerciasis endemicity status per ESPEN, and which countries were included in our geospatial analysis from 2000 to 2018. Endemicity classifications were derived from ESPEN data at the level of intervention units (IU) (retrieved 14 February 2020). Countries outside the WHO AFRO region are not covered by the scope of ESPEN; countries in the WHO EMRO (Eastern Mediterranean Regional Office) region are listed here if they are in Africa or have evidence of onchocerciasis endemicity or uncertain status. We considered countries endemic if they had at least one intervention unit that was flagged as endemic by ESPEN. Number of observations: The number of data rows in the final dataset for a given country.

| **Location** | **ESPEN** **endemicity** | **Included in 2000–2018 model?** | **Number of Observations** |
| --- | --- | --- | --- |
| ***AFRO***(1) |  |  |  |
| Algeria | Non-endemic | no | - |
| Angola | Endemic | yes | 876 |
| Benin | Endemic | yes | 300 |
| Botswana | Non-endemic | no | - |
| Burkina Faso | Endemic | yes | 72 |
| Burundi | Endemic | yes | 263 |
| Cabo Verde | Non-endemic | no | - |
| Cameroon | Endemic | yes | 1216 |
| Central African Republic | Endemic | yes | 1079 |
| Chad | Endemic | yes | 753 |
| Comoros | Non-endemic | no | - |
| Republic of the Congo | Endemic | yes | 490 |
| Côte d’Ivoire | Endemic | yes | 95 |
| Democratic Republic of the Congo | Endemic | yes | 4574 |
| Equatorial Guinea | Endemic | yes | 341 |
| Eritrea | Non-endemic | no | - |
| Eswatini | Non-endemic | no | - |
| Ethiopia | Endemic | yes | 1062 |
| Gabon | Endemic | yes | 157 |
| The Gambia | Non-endemic | yes | 0 |
| Ghana | Endemic | yes | 78 |
| Guinea | Endemic | yes | 193 |
| Guinea-Bissau | Endemic | yes | 0 |
| Kenya | Consider oncho elimination mapping | yes | 94 |
| Lesotho | Non-endemic | no | - |
| Liberia | Endemic | yes | 114 |
| Madagascar | Non-endemic | no | - |
| Malawi | Endemic | yes | 333 |
| Mali | Endemic | yes | 160 |
| Mauritania | Non-endemic | no | - |
| Mauritius | Mapping gap (one district) | no | - |
| Mozambique | Endemic | yes | 291 |
| Namibia | Consider oncho elimination mapping (some districts) | no | - |
| Niger | Endemic | yes | 0 |
| Nigeria | Endemic | yes | 3445 |
| Rwanda | Consider oncho elimination mapping | yes | 90 |
| São Tomé and Príncipe | Non-endemic | yes | 0 |
| Senegal | Endemic | yes | 67 |
| Seychelles | Non-endemic | no | - |
| Sierra Leone | Endemic | yes | 223 |
| South Africa | Non-endemic | no | - |
| South Sudan | Endemic | yes | 466 |
| Togo | Endemic | yes | 160 |
| Uganda | Endemic | yes | 620 |
| Tanzania | Endemic | yes | 478 |
| Zambia | Consider oncho elimination mapping (some districts) | no | - |
| Zimbabwe | Non-endemic | no | - |
|  |  |  |  |
| ***EMRO***(2) |  |  |  |
| Djibouti | - | no | - |
| Egypt | - | no | - |
| Libya | - | no | - |
| Morocco | - | no | - |
| Somalia | - | yes | 0 |
| Sudan | (Endemic) | yes | 26 |
| Tunisia | - | no | - |
| Yemen | (Endemic) | yes | 0 |

## **3.2 Systematic review**

Articles related to onchocerciasis were found by searching PubMed, Scopus, and Web of Science using the following keywords: “Oncho”, “river blindness”, “O. Volvulus”, “robles disease”, “blinding filariasis”, “coast erysipelas”, and “sowda”. A systematic review of these reports, all published before July 7, 2017, was then conducted. A second round of formal reviews was undertaken on June 6, 2019, to cover new articles published after July 7, 2017, and followed the same process as the first round.

### 3.2.1 Systematic review data processing

The systematic review process is illustrated in Supplementary Figure 2. Throughout the systematic review we excluded publications that met the following criteria: no measurement of onchocerciasis prevalence, data collected before 1985, case-control studies, qualitative research publications, duplicative data from cohort studies, hospital-based studies, and publications that did not report the location of data collection. The search identified 4048 publications, which were reduced to 579 after screening titles and abstracts. A full-text review was completed for the remaining publications. The full-text review yielded 259 publications which met the inclusion criteria and were extracted. The literature review was updated on June 6, 2019, by searching PubMed with the same search string used in 2017 for articles published after July 7, 2017. The search returned 304 results, which were narrowed to 115 articles after screening titles and abstracts. A full-text review resulted in 22 articles that were eligible for extraction. Additional publications were identified outside of the literature review and screened for inclusion by the same criteria, up through April 14, 2020. The final dataset drew from 290 articles. Overall, 16 096 datapoints were extracted and geo-located from 290 publications. Data extracted from each source included survey year, age-range of individuals tested, diagnostic information, sample size, number of individuals tested positive, and sampling strategy details.

Supplementary Figure 2. Onchocerciasis article review and data extraction flowchart.

Each step of the extraction process is outlined, from article identification and screening to extraction, including the number of articles or records that were processed or removed in each step before reaching the final dataset. Additional articles outside of the literature review were identified and screened for inclusion (“ongoing process”) on an ongoing basis up until April 14, 2020.

## **3.3 Geo-positioning**

Geographical information associated with onchocerciasis prevalence data were verified and geo-located to ensure accuracy. Data associated with locations smaller than 5 × 5 km were treated as points and geo-located as latitude/longitude coordinates. Coordinates provided by a data source were mapped to ensure that the coordinates were located in the correct administrative units. If coordinates were not reported, points were geolocated and vetted by comparing results from Google Maps, Fuzzy Gazetteer, Geonames, and Open Street Map. Locations larger than 5 × 5 km were treated as polygon data and geo-located to the most granular administrative boundary possible (most commonly district level). Custom shapefiles were created to geolocate areas that did not align with administrative boundaries. In the event that a literature source only included a map of locations sampled without any other information, ArcGIS software was utilised to overlay the map onto existing administrative boundaries, and location coordinates or custom polygons were manually created and recorded. Prevalence data that were reported for administrative areas were matched to their appropriate polygon by searching our administrative shapefile database. If place names were unidentifiable across multiple shapefile libraries or geo-referencing sources, they were excluded from the analysis.

## **3.4 Data processing**

Data were excluded from the analysis for the following reasons: did not report survey year (N = 8236); did not report post-1988 data (N = 2648); did not report skin snip microscopy or nodule prevalence (N = 12 121); did not report sample size or prevalence (N = 30); or did not report data for countries in the modelling region or could not be accurately georeferenced (N = 580). Duplicate records were identified and excluded (N = 1592); these generally reflected different data sources (eg, literature extraction and ESPEN) which contained the same survey, as judged by identical year, location, sample size, cases, and reference when available, although there was some deduplication within individual data sources. Prior to polygon resampling (see section 5.2), the dataset consisted of 18 116 georeferenced prevalence observations, consisting of 17 896 point-referenced observations and 220 areal observations. A total of 14 314 observations represented prevalence based on nodule examinations, and 3802 observations represented microfiladermia prevalence as measured by skin snip assays. After resampling, the full modelling dataset consisted of 20 124 georeferenced datapoints.

Supplementary Table 3 provides citations for data sources used in our onchocerciasis MBG model. The geographical coverage of the final dataset is summarised in Supplementary Figure 3.

**Supplementary Table 3. Citations for data inputs.**

The NID is a unique identifier cataloguing all data inputs in the Global Health Data Exchange (<http://ghdx.healthdata.org>). Note: Records are listed here in alphabetical order by geography, but some sources provided data for multiple countries; such sources are listed here only once.

| **NID** | **Geographies** | **Citation** |
| --- | --- | --- |
| 332798 | Angola | Carme B, Ntsoumou-Madzou V, Samba Y, Yebakima A. Prevalence of depigmentation of the shins: a simple and cheap way to screen for severe endemic onchocerciasis in Africa. Bull World Health Organ. 1993; 71(6): 755–8. |
| 334477 | Benin | Gallin M, Adams A, Kruppa TF, Gbaguidi EA, Massougbodji A, Sadeler BC, Brattig N, Erttmann KD. Epidemiological studies of onchocerciasis in southern Benin. Trop Med Parasitol. 1993; 44(2): 69–74. |
| 125405 | Burkina Faso | Kabore JK, Cabore JW, Melaku Z, Druet-Cabanac M, Preux PM. Epilepsy in a focus of onchocerciasis in Burkina Faso. Lancet. 1996; 347(9004): 836. |
| 334475 | Burkina Faso | De Sole G, Remme J. Importance of migrants infected with Onchocerca volvulus in west African river valleys protected by 14 to 15 years of Simulium control. Trop Med Parasitol. 1991; 42(2): 75–8. |
| 332903 | Burkina Faso, Côte d’Ivoire | Toè L, Adjami AG, Boatin BA, Back C, Alley ES, Dembélé N, Brika PG, Pearlman E, Unnasch TR. Topical application of diethylcarbamazine to detect onchocerciasis recrudescence in west Africa. Trans R Soc Trop Med Hyg. 2000; 94(5): 519–25. |
| 334481 | Burundi | Newell E d. Comparison of the use of skin scarification and skin biopsies to determine the prevalence and intensity of Onchocerca volvulus infection. Ann Trop Med Parasitol. 1997; 91(6): 633. |
| 338571 | Burundi | Newell ED, Hicuburundi B, Ndimuruvugo N. [Endemicity and clinical manifestations of onchocerciasis in the province of Bururi, Burundi]. Trop Med Int Health. 1997; 2(3): 218–26. |
| 338573 | Burundi | Newell ED, Ndimuruvugo N, Nimpa D. [Endemicity and clinical manifestations of onchocerciasis in the provinces of Cibitoke and Bubanza (Burundi)]. Bull Soc Pathol Exot. 1997; 90(5): 353–7. |
| 136492 | Cameroon | Cho-Ngwa F, Amambua AN, Ambele MA, Titanji VPK. Evidence for the exacerbation of lymphedema of geochemical origin, podoconiosis, by onchocerciasis. J Infect Public Health. 2009; 2(4): 198-203. |
| 159316 | Cameroon | Matthews GA, Dobson HM, Nkot PB, Wiles TL, Birchmore M. Preliminary examination of integrated vector management in a tropical rainforest area of Cameroon. Trans R Soc Trop Med Hyg. 2009; 103(11): 1098-104. |
| 324729 | Cameroon | Kamga G-R, Dissak-Delon FN, Nana-Djeunga HC, Biholong BD, Mbigha-Ghogomu S, Souopgui J, Zoure HGM, Boussinesq M, Kamgno J, Robert A. Still mesoendemic onchocerciasis in two Cameroonian community-directed treatment with ivermectin projects despite more than 15 years of mass treatment. Parasites Vectors. 2016; 9(1): 581. |
| 327960 | Cameroon | Wanji S, Kengne-Ouafo JA, Esum ME, Chounna PWN, Tendongfor N, Adzemye BF, Eyong JEE, Jato I, Datchoua-Poutcheu FR, Kah E, Enyong P, Taylor DW. Situation analysis of parasitological and entomological indices of onchocerciasis transmission in three drainage basins of the rain forest of South West Cameroon after a decade of ivermectin treatment. Parasites Vectors. 2015; 8: 202. |
| 327968 | Cameroon | Katabarwa MN, Eyamba A, Chouaibou M, Enyong P, Kuété T, Yaya S, Yougouda A, Baldiagaï J, Madi K, Andze GO, Richards F. Does onchocerciasis transmission take place in hypoendemic areas? a study from the North Region of Cameroon. Trop Med Int Health. 2010; 15(5): 645-52. |
| 327992 | Cameroon | Katabarwa MN, Eyamba A, Nwane P, Enyong P, Kamgno J, KuetÃ© T, Yaya S, Aboutou R, Mukenge L, Kafando C, Siaka C, Mkpouwoueiko S, Ngangue D, Biholong BD, Andze GO. Fifteen years of annual mass treatment of onchocerciasis with ivermectin have not interrupted transmission in the west region of cameroon. J Parasitol Res. 2013; 2013: 420928. |
| 328065 | Cameroon | Katabarwa MN, Eyamba A, Nwane P, Enyong P, Yaya S, BaldiagaÃ¯ J, Madi TK, Yougouda A, Andze GO, Richards FO. Seventeen years of annual distribution of ivermectin has not interrupted onchocerciasis transmission in North Region, Cameroon. Am J Trop Med Hyg. 2011; 85(6): 1041-9. |
| 328095 | Cameroon | Kamga HLF, Shey DN, Assob JCN, Njunda AL, Nde Fon P, Njem PK. Prevalence of onchocerciasis in the Fundong Health District, Cameroon after 6 years of continuous community-directed treatment with ivermectin. Pan Afr Med J. 2011; 10: 34. |
| 332745 | Cameroon | Pion SDS, Clarke P, Filipe J a. N, Kamgno J, Gardon J, Basáñez M-G, Boussinesq M. Co-infection with Onchocerca volvulus and Loa loa microfilariae in central Cameroon: are these two species interacting?. Parasitology. 2006; 132(Pt 6): 843–54. |
| 332783 | Cameroon | Boussinesq M, Chippaux JP, Ernould JC, Quillevere D, Prod’hon J. Effect of repeated treatments with ivermectin on the incidence of onchocerciasis in northern Cameroon. Am J Trop Med Hyg. 1995; 53(1): 63–7. |
| 332786 | Cameroon | Kollo B, Mather FJ, Cline BL. Evaluation of alternate methods of rapid assessment of endemicity of Onchocerca volvulus in communities in southern Cameroon. Am J Trop Med Hyg. 1995; 53(3): 243–7. |
| 332788 | Cameroon | Ngoumou P, Walsh JF, Mace JM. A rapid mapping technique for the prevalence and distribution of onchocerciasis: a Cameroon case study. Ann Trop Med Parasitol. 1994; 88(5): 463–74. |
| 332790 | Cameroon | Mendoza Aldana J, Piechulek H, Maguire J. Forest onchocerciasis in Cameroon: its distribution and implications for selection of communities for control programmes. Ann Trop Med Parasitol. 1997; 91(1): 79–86. |
| 332840 | Cameroon | Ayong LS, Tume CB, Wembe FE, Simo G, Asonganyi T, Lando G, Ngu JL. Development and evaluation of an antigen detection dipstick assay for the diagnosis of human onchocerciasis. Trop Med Int Health. 2005; 10(3): 228-33. |
| 332848 | Cameroon | Wanji S, Tendongfor N, Esum M, Ndindeng S, Enyong P. Epidemiology of concomitant infections due to Loa loa, Mansonella perstans, and Onchocerca volvulus in rain forest villages of Cameroon. Med Microbiol Immunol. 2003; 192(1): 15-21. |
| 332893 | Cameroon | Kamgno J, Boussinesq M. [Hyperendemic loaiasis in the Tikar plain, shrub savanna region of Cameroon]. Bull Soc Pathol Exot. 2001; 94(4): 342–6. |
| 332897 | Cameroon | Esum M, Wanji S, Tendongfor N, Enyong P. Co-endemicity of loiasis and onchocerciasis in the South West Province of Cameroon: implications for mass treatment with ivermectin. Trans R Soc Trop Med Hyg. 2001; 95(6): 673–6. |
| 338584 | Cameroon | Kamgno J, Bouchité B, Baldet T, Folefack G, Godin C, Boussinesq M. [Study of the distribution of human filariasis in West Province of Cameroon]. Bull Soc Pathol Exot. 1997; 90(5): 327–30. |
| 332820 | Cameroon, Central African Republic, Gabon | Ozoh G, Boussinesq M, Bissek A-CZ-K, Kobangue L, Kombila M, Mbina J-RM, Enyong P, Noma M, Sékétéli A, Fobi G. Evaluation of the diethylcarbamazine patch to evaluate onchocerciasis endemicity in Central Africa. Trop Med Int Health. 2007; 12(1): 123-9. |
| 327872 | Cameroon, Nigeria, Sudan, Uganda | Ozoh GA, Murdoch ME, Bissek A-C, Hagan M, Ogbuagu K, Shamad M, Braide EI, Boussinesq M, Noma MM, Murdoch IE, Sékétéli A, Amazigo UV. The African Programme for Onchocerciasis Control: impact on onchocercal skin disease. Trop Med Int Health. 2011; 16(7): 875–83. |
| 332855 | Central African Republic | Kennedy MH, Bertocchi I, Hopkins AD, Meredith SE. The effect of 5 years of annual treatment with ivermectin (Mectizan) on the prevalence and morbidity of onchocerciasis in the village of Gami in the Central African Republic. Ann Trop Med Parasitol. 2002; 96(3): 297-307. |
| 332796 | Congo | Brito M, Paulo R, Van-Dunem P, Martins A, Unnasch TR, Novak RJ, Jacob B, Stanton MC, Molyneux DH, Kelly-Hope LA. Rapid Integrated Clinical Survey to Determine Prevalence and Co-distribution Patterns of Lymphatic Filariasis and Onchocerciasis in a Loa Loa Co-endemic Area: The Angolan Experience. Parasite Epidemiol Control. 2017; 2(3): 71-84. |
| 338569 | Congo | Talani P, Missamou F, Kankou JM, Niabe B, Obengui null, Moyen G. [Rapid epidemiological mapping of onchocerciasis in Congo Brazzaville]. Dakar Med. 2005; 50(3): 164–7. |
| 327962 | Côte d’Ivoire | Lloyd MM, Gilbert R, Taha NT, Weil GJ, Meite A, Kouakou IMM, Fischer PU. Conventional parasitology and DNA-based diagnostic methods for onchocerciasis elimination programmes. Acta Trop. 2015; 146: 114-8. |
| 338588 | Côte d’Ivoire | Diarrassouba S, Traore S, Riviere F. [Endemic onchocerciasis in forested zones of Ivory Coast: prevalence rate and microfilarial densities]. Med Trop (Mars). 1996; 56(1): 59–62. |
| 332853 | Democratic Republic of Congo | Kayembe DL, Kasonga DL, Kayembe PK, Mwanza J-CK, Boussinesq M. Profile of eye lesions and vision loss: a cross-sectional study in Lusambo, a forest-savanna area hyperendemic for onchocerciasis in the Democratic Republic of Congo. Trop Med Int Health. 2003; 8(1): 83-9. |
| 327885 | Ethiopia | Feleke SM, Tadesse G, Mekete K, Tekle AH, Kebede A. Epidemiological Mapping of Human Onchocerciasis in Transmission Suspected Districts of Bale, Borena, and West Arsi Zones of Eastern Ethiopia. Interdiscip Perspect Infect Dis. 2016; 2016: 6937509. |
| 327957 | Ethiopia | Dana D, Debalke S, Mekonnen Z, Kassahun W, Suleman S, Getahun K, Yewhalaw D. A community-based cross-sectional study of the epidemiology of onchocerciasis in unmapped villages for community directed treatment with ivermectin in Jimma Zone, southwestern Ethiopia. BMC Public Health. 2015; 15: 595. |
| 328057 | Ethiopia | Dori GU, Belay T, Belete H, Panicker KN, Hailu A. Parasitological and clinico-epidemiological features of onchocerciasis in West Wellega, Ethiopia. J Parasit Dis. 2012; 36(1): 10-8. |
| 332186 | Ethiopia | Mengistu G, Balcha F, Britton S. Co-infection of Onchocerca volvulus and intestinal helminths in indigenous and migrant farmers in southwest Ethiopia. Ethiop Med J. 2002; 40(1): 19–27. |
| 332189 | Ethiopia | Jira C. Prevalence of onchocerciasis in Blue Nile valley of western Ethiopia. Indian J Public Health. 1993; 37(4): 135–7. |
| 332193 | Ethiopia | Taye A, Gebre-Michael T, Taticheff S. Onchocerciasis in Gilgel Ghibe River Valley southwest Ethiopia. East Afr Med J. 2000; 77(2): 116–20. |
| 332674 | Ethiopia | Adugna N, Woldegiorgis M, Tilahun D, Kebede A, Hadis M. The Epidemiology of Onchocerciasis in the Resettled and Indigenous Population in Pawe, Western Ethiopia. Ethiop Med J. 1999; 37(1): 41-50. |
| 332676 | Ethiopia | Legesse M, Balcha F, Erko B. Status of onchocerciasis in Teppi area, Southwestern Ethiopia, after four years of annual community-directed treatment with ivermectin. Ethiop J Health Dev. 2010; 24(1): 51-56. |
| 332678 | Ethiopia | Samuel A, Belay T, Yehalaw D, Taha M, Zemene E, Zeynudin A. Impact of Six Years Community Directed Treatment with Ivermectin in the Control of Onchocerciasis, Western Ethiopia. PLoS One. 2016; 11(3): e0141029. |
| 332851 | Ethiopia | Hailu A, Balcha F, Birrie H, Berhe N, Aga A, Mengistu G, Bezuneh A, Ali A, Gebre-Michael T, Gemetchu T. Prevalence of onchocercal skin disease and infection among workers of coffee plantation farms in Teppi, southwestern Ethiopia. Ethiop Med J. 2002; 40(3): 259-69. |
| 332825 | Gabon | Fobi G, Mourou Mbina JR, Ozoh G, Kombila M, Agaya C, Olinga Olinga JM, Boussinesq M, Enyong P, Noma M, Sékétéli A. [Onchocerciasis in the area of Lastourville, Gabon. Clinical and entomological aspects]. Bull Soc Pathol Exot. 2006; 99(4): 269-71. |
| 327974 | Ghana | Garms R, Badu K, Owusu-Dabo E, Baffour-Awuah S, Adjei O, Debrah AY, Nagel M, Biritwum NK, Gankpala L, Post RJ, Kruppa TF. Assessments of the transmission of Onchocerca volvulus by Simulium sanctipauli in the Upper Denkyira District, Ghana, and the intermittent disappearance of the vector. Parasitol Res. 2015; 114(3): 1129-37. |
| 327912 | Ghana, Sri Lanka | Frempong KK, Walker M, Cheke RA, Tetevi EJ, Gyan ET, Owusu EO, Wilson MD, Boakye DA, Taylor MJ, Biritwum N-K, Osei-Atweneboana M, Basáñez M-G. Does Increasing Treatment Frequency Address Suboptimal Responses to Ivermectin for the Control and Elimination of River Blindness?. Clin Infect Dis. 2016; 62(11): 1338–47. |
| 332662 | Malawi, Tanzania | Courtright P, Johnston K, Chitsulo L. A new focus of onchocerciasis in Mwanza District, Malawi. Trans R Soc Trop Med Hyg. 1995; 89(1): 34–6. |
| 144330 | Mali, Senegal | Diawara L, Traoré MO, Badji A, Bissan Y, Doumbia K, Goita SF, Konaté L, Mounkoro K, Sarr MD, Seck AF, Toé L, Tourée S, Remme JH. Feasibility of onchocerciasis elimination with ivermectin treatment in endemic foci in Africa: first evidence from studies in Mali and Senegal. PLoS Negl Trop Dis. 2009; 3(7): e497. |
| 191825 | Mali, Senegal | Traore MO, Sarr MD, Badji A, Bissan Y, Diawara L, Doumbia K, Goita SF, Konate L, Mounkoro K, Seck AF, Toe L, Toure S, Remme JH. Proof-of-principle of onchocerciasis elimination with ivermectin treatment in endemic foci in Africa: final results of a study in Mali and Senegal. PLoS Negl Trop Dis. 2012; 6(9): e1825. |
| 136520 | Nigeria | Anosike JC, Onwuliri CO. Studies on filariasis in Bauchi State, Nigeria. II. The prevalence of human filariasis in Darazo Local Government area. Appl Parasitol. 1994; 35(4): 242-50. |
| 147655 | Nigeria | Anosike JC, Onwuliri COE, Onwuliri VA. Human filariasis in Dass local government area of Bauchi state, Nigeria. Trop Ecol. 2003; 44(2): 217-27. |
| 147869 | Nigeria | Uttah E, Ibeh DC. Multiple filarial species microfiladermia: a comparative study of areas with endemic and sporadic onchocerciasis. J Vector Borne Dis. 2011; 48(4): 197-204. |
| 324133 | Nigeria | Evans DS, Alphonsus K, Umaru J, Eigege A, Miri E, Mafuyai H, Gonzales-Peralta C, Adamani W, Pede E, Umbugadu C, Saka Y, Okoeguale B, Richards FO. Status of Onchocerciasis transmission after more than a decade of mass drug administration for onchocerciasis and lymphatic filariasis elimination in central Nigeria: challenges in coordinating the stop MDA decision. PLoS Negl Trop Dis. 2014; 8(9): 1-10. |
| 327876 | Nigeria | Eyo J, Onyishi G, Ugokwe C. Rapid epidemiological assessment of onchocerciasis in a tropical semi-urban community, enugu state, Nigeria. Iran J Parasitol. 2013; 8(1): 145–51. |
| 327921 | Nigeria | Katabarwa M, Eyamba A, Habomugisha P, Lakwo T, Ekobo S, Kamgno J, Kuete T, Ndyomugyenyi R, Onapa A, Salifou M, Ntep M, Richards FO. After a decade of annual dose mass ivermectin treatment in Cameroon and Uganda, onchocerciasis transmission continues. Trop Med Int Health. 2008; 13(9): 1196-1203. |
| 327941 | Nigeria | Okoye IC, Onwuliri CO. Epidemiology and psycho-social aspects of onchocercal skin diseases in northeastern Nigeria. Filaria J. 2007; 6: 15. |
| 327954 | Nigeria | Okoye IC, Onwuliri CO. Epidemiology and psycho-social aspects of onchocercal skin diseases in northeastern Nigeria. Filaria J. 2007; 6: 15. |
| 327966 | Nigeria | Ojurongbe O, Akindele AA, Adeleke MA, Oyedeji MO, Adedokun SA, Ojo JF, Akinleye CA, Bolaji OS, Adefioye OA, Adeyeba OA. Co-endemicity of loiasis and onchocerciasis in rain forest communities in southwestern Nigeria. PLoS Negl Trop Dis. 2015; 9(3): e0003633. |
| 327972 | Nigeria | Opara KN, Fagbemi BO. Population dynamics of onchocerca volvulus microfilariae in human host after six years of drug control. J Vector Borne Dis. 2008; 45(1): 29-37. |
| 327982 | Nigeria | Akinbo FO, Okaka CE. Hyperendemicity of onchocerciasis in Ovia Northeast Local Government Area, Edo State, Nigeria. East Afr J Public Health. 2010; 7(1): 84-6. |
| 328059 | Nigeria | Osue HO, Inabo HI, Yakubu SE, Audu PA, Galadima M, Odama LE, Musa D, Ado SA, Mamman M. Impact of Eighteen-Year Varied Compliance to Onchocerciasis Treatment with Ivermectin in Sentinel Savannah Agrarian Communities in Kaduna State of Nigeria. ISRN Parasitol. 2013; 2013: 960168. |
| 328063 | Nigeria | Tekle AH, Elhassan E, Isiyaku S, Amazigo UV, Bush S, Noma M, Cousens S, Abiose A, Remme JH. Impact of Long-term Treatment of Onchocerciasis with Ivermectin in Kaduna State, Nigeria: First Evidence of the Potential for Elimination in the Operational Area of the African Programme for Onchocerciasis Control. Parasites Vectors. 2012; 5(1). |
| 328083 | Nigeria | Uttah E. Onchocerciasis in the upper imo river basin, Nigeria: prevalence and comparative study of waist and shoulder snips from mesoendemic communities. Iran J Parasitol. 2010; 5(2): 33-41. |
| 328089 | Nigeria | Sam-Wobo SO, Adeleke MA, Jayeola OA, Adeyi AO, Oluwole AS, Ikenga M, Lawniye F, Gazama J, Kagni A, Kosoko TO, Agbeyangi O, Bankole S, Toé L, Mafiana CF, Yameogo L. Epidemiological evaluation of onchocerciasis along Ogun River System, southwest Nigeria. J Vector Borne Dis. 2012; 49(2): 101-4. |
| 332571 | Nigeria | Akogun OB, Akoh JI, Hellandendu H. Non-ocular clinical onchocerciasis in relation to skin microfilaria in the Taraba River Valley, Nigeria. J Hyg Epidemiol Microbiol Immunol. 1992; 36(4): 368–83. |
| 332576 | Nigeria | Anosike JC, Onwuliri CO. Studies on filariasis in Bauchi State, Nigeria. 1. Endemicity of human onchocerciasis in Ningi Local Government Area. Ann Trop Med Parasitol. 1995; 89(1): 31–8. |
| 332594 | Nigeria | Nwaorgu OC, Ohaegbula A, Onweluzo IE, Alo ET, Nweke LN, Agu ML, Emeh E. Results of a large scale onchocercosis survey in Enugu State, Nigeria. J Helminthol. 1994; 68(2): 155–9. |
| 332703 | Nigeria | Emukah E, Nimzing J, Ezeabikwa M, Obiezu J, Okpala N, Miri E, Katabarwa M, Hopkins D, Richards F. Impact assessment of repeated annual ivermectin on ocular and clinical onchocerciasis 14 years of annual mass drug administration in eight sentinel villages, southeast Nigeria 2008. Am J Trop Med Hyg. 2010; 83(Suppl 5): 22. |
| 332707 | Nigeria | Olofintoye LK, Adewole SO, Erinle FB, Ajayi AI. Prevalence of Onchoceriasis Syndrome in Ise-Orun Local Government Area of Ekiti State, Nigeria. Pak J Sci Ind Res. 2005; 48(6): 412-6. |
| 332712 | Nigeria | Oyibo WA, Fagbenro-Beyioku AF. Reduced prevalence of onchocerciasis following mass treatment with ivermectin. East Afr Med J. 1997; 74(5): 326–30. |
| 332714 | Nigeria | Rebecca SN, Akinboye DO, Abdulazeez AA. Onchocerciasis and Plasmodiasis: Concurrent Infection in Garaha-Dutse Community, Adamawa State Nigeria. Biomed Res. 2008; 19(2): 107-11. |
| 332717 | Nigeria | Abanobi OC, Anosike JC. Control of onchocerciasis in Nzerem-Ikpem, Nigeria: baseline prevalence and mass distribution of ivermectin. Public Health. 2000; 114(5): 402–6. |
| 332719 | Nigeria | Akinboye DO, Okwong E, Ajiteru N, Fawole O, Agbolade O, Ayinde OO, Amosu AM, Atulomah NOS, Oduola O, Owodunni BM, Rebecca SN, Falade M. Onchocerciasis Among Inhabitants of Ibarapa Local Government Community of Oyo State, Nigeria. Biomed Res. 2010; 21(2): 174-8. |
| 332723 | Nigeria | Akogun OB, Musa-Hong H, Hellandendu H. Onchocerciasis in Taraba State, Nigeria: intensity, rate of infection and associated symptoms in 14 communities of Bali district. Appl Parasitol. 1994; 35(2): 125–32. |
| 332725 | Nigeria | Akogun OB, Akoh JI, Okolo A. Comparison of two sample survey methods for hyperendemic onchocerciasis and a new focus in Dakka, Nigeria. Rev Biol Trop. 1997; 45(2): 871–6. |
| 332728 | Nigeria | Akogun OB. Onchocerciasis in Taraba State, Nigeria: clinical-epidemiological study of at-risk males in Bakundi District. Zentralbl Bakteriol. 1999; 289(3): 371–9. |
| 332730 | Nigeria | Nmorsi OPG, Oladokun I a. A, Egwunyenga OA, Oseha E. Eye lesions and onchocerciasis in a rural farm settlement in Delta state, Nigeria. Southeast Asian J Trop Med Public Health. 2002; 33(1): 28–32. |
| 332732 | Nigeria | Opara KN, Fagbemi BO, Atting IA, Oyene UE, Okenu DMN. Status of forest onchocerciasis in the Lower Cross River Basin, Nigeria: change in clinical and parasitological indices after 6 years of ivermectin intervention. Public Health. 2007; 121(3): 202–7. |
| 332734 | Nigeria | Umeh RE, Chijioke CP, Okonkwo PO. Eye disease in an onchocerciasis-endemic area of the forest-savanna mosaic region of Nigeria. Bull World Health Organ. 1996; 74(1): 95–100. |
| 332736 | Nigeria | Umeh RE. The causes and profile of visual loss in an onchocerciasis-endemic forest-savanna zone in Nigeria. Ophthalmic Epidemiol. 1999; 6(4): 303–15. |
| 332899 | Nigeria | Anosike JC, Celestine NA, Onwuliri OE, Onwuliri VA. The prevalence, intensity and clinical manifestations of Onchocerca volvulus infection in Toro local government area of Bauchi State, Nigeria. Int J Hyg Environ Health. 2001; 203(5–6): 459–64. |
| 332905 | Nigeria | Nwosu SN. Low vision in persons aged 50 and above in the onchocercal endemic communities of Anambra State, Nigeria. West Afr J Med. 2000; 19(3): 216–9. |
| 332907 | Nigeria | Abanobi OC. Cross-validation of the rapid epidemiological mapping of onchocerciasis endemicity in Anambra state, Nigeria. Ann Trop Med Parasitol. 1999; 93(7): 721–6. |
| 327879 | Senegal | Wilson NO, Badara Ly A, Cama VA, Cantey PT, Cohn D, Diawara L, Direny A, Fall M, Feeser KR, Fox LM, Kabore A, Seck AF, Sy N, Ndiaye D, Dubray C. Evaluation of Lymphatic Filariasis and Onchocerciasis in Three Senegalese Districts Treated for Onchocerciasis with Ivermectin. PLoS Negl Trop Dis. 2016; 10(12): e0005198. |
| 136415 | Sierra Leone | Gbakima AA, Sahr F. Filariasis in the Kaiyamba Chiefdom, Moyamba District Sierra Leone: an epidemiological and clinical study. Public Health. 1996; 110(3): 169-74. |
| 155864 | Sierra Leone | Gbakima AA. Inland valley swamp rice development: malaria, schistosomiasis, onchocerciasis in south central Sierra Leone. Public Health. 1994; 108(2): 149-57. |
| 334430 | Sierra Leone | Gbakima AA, Barbe RF. Onchocerca Volvulus infection in Sierra Leone: relation between prevalence, intensity of infection, and ocular problems in a “forest” region. Acta Leiden. 1992; 60(2): 47–59. |
| 334432 | Sierra Leone | Gbakima AA. Integrated control of Onchocerca volvulus infection in a hyperendemic zone in Sierra Leone. East Afr Med J. 1996; 73(3): 159–63. |
| 327874 | Sudan | Higazi TB, Zarroug IMA, Mohamed HA, Elmubark WA, Deran TCM, Aziz N, Katabarwa M, Hassan HK, Unnasch TR, Mackenzie CD, Richards F, Hashim K. Interruption of Onchocerca volvulus transmission in the Abu Hamed focus, Sudan. Am J Trop Med Hyg. 2013; 89(1): 51–7. |
| 327986 | Tanzania | Mweya CN, Kalinga AK, Kabula B, Malley KD, Ruhiso MH, Maegga BTA. Onchocerciasis situation in the Tukuyu focus of southwest Tanzania after ten years of ivermectin mass treatment. Tanzan Health Res Bull. 2007; 9(3): 174-9. |
| 332901 | Tanzania | Makunde WH, Salum FM, Massaga JJ, Alilio MS. Clinical and parasitological aspects of itching caused by onchocerciasis in Morogoro, Tanzania. Ann Trop Med Parasitol. 2000; 94(8): 793–9. |
| 327919 | Togo | Golden A, Faulx D, Kalnoky M, Stevens E, Yokobe L, Peck R, Karabou P, Banla M, Rao R, Adade K, Gantin RG, Komlan K, Soboslay PT, de Los Santos T, Domingo GJ. Analysis of age-dependent trends in Ov16 IgG4 seroprevalence to onchocerciasis. Parasites Vectors. 2016; 9(1): 338. |
| 338602 | Togo | Dogba PK, Komi BT. [The evaluation of endemic onchocerciasis in the Amou prefecture (Togo)]. Bull Soc Pathol Exot. 1994; 87(2): 110–1. |
| 327978 | Uganda | Katabarwa M, Lakwo T, Habomugisha P, Agunyo S, Byamukama E, Oguttu D, Ndyomugyenyi R, Tukesiga E, Ochieng GO, Abwaimo F, Onapa A, Lwamafa DWK, Walsh F, Unnasch TR, Richards FO. Transmission of Onchocerca volvulus by Simulium neavei in Mount Elgon focus of Eastern Uganda has been interrupted. Am J Trop Med Hyg. 2014; 90(6): 1159-66. |
| 328051 | Uganda | Katabarwa MN, Lakwo T, Habomugisha P, Agunyo S, Byamukama E, Oguttu D, Tukesiga E, Unoba D, Dramuke P, Onapa A, Tukahebwa EM, Lwamafa D, Walsh F, Unnasch TR. Transmission of Onchocerca volvulus continues in Nyagak-Bondo focus of northwestern Uganda after 18 years of a single dose of annual treatment with ivermectin. Am J Trop Med Hyg. 2013; 89(2): 293-300. |
| 332836 | Uganda | Kipp W, Bamhuhiiga J, Rubaale T, Kabagambe G. Adverse reactions to the ivermectin treatment of onchocerciasis patients: does infection with the human immunodeficiency virus play a role?. Ann Trop Med Parasitol. 2005; 99(4): 395-402. |
| 332845 | Uganda | Ndyomugyenyi R, Tukesiga E, Büttner DW, Garms R. The impact of ivermectin treatment alone and when in parallel with Simulium neavei elimination on onchocerciasis in Uganda. Trop Med Int Health. 2004; 9(8): 882-6. |
| 333505 | Uganda | Fischer P, Kipp W, Bamuhiga J, Binta-Kahwa J, Kiefer A, Büttner DW. Parasitological and clinical characterization of Simulium neavei-transmitted onchocerciasis in western Uganda. Trop Med Parasitol. 1993; 44(4): 311–21. |
| 333506 | Uganda | Fischer P, Kipp W, Kabwa P, Buttner DW. Onchocerciasis and human immunodeficiency virus in western Uganda: prevalences and treatment with ivermectin. Am J Trop Med Hyg. 1995; 53(2): 171–8. |
| 334413 | Uganda | Katabarwa M, Onapa AW, Nakileza B. Rapid epidemiological mapping of onchocerciasis in areas of Uganda where Simulium neavei sl is the vector. East Afr Med J. 1999; 76(8): 440–6. |
| 334415 | Uganda | Katabarwa MN, Walsh F, Habomugisha P, Lakwo TL, Agunyo S, Oguttu DW, Unnasch TR, Unoba D, Byamukama E, Tukesiga E, Ndyomugyenyi R, Richards FO. Transmission of Onchocerciasis in Wadelai Focus of Northwestern Uganda Has Been Interrupted and the Disease Eliminated [Internet]. J Parasitol Res. 2012. |
| 334417 | Uganda | Katabarwa M. Has interruption of Simulium neavei s.s transmitted onchocerciasis been attained in the Kashoya-Kitomi focus?. 2010. |
| 334419 | Uganda | Kipp W, Bamuhiga JT, Kwered EM. Onchocerciasis prevalence in previously known foci in western Uganda: results from a preliminary survey in Kabarole district. Trop Med Parasitol. 1992; 43(2): 80–2. |
| 334420 | Uganda | Lakwo TL, Garms R, Rubaale T, Katabarwa M, Walsh F, Habomugisha P, Oguttu D, Unnasch T, Namanya H, Tukesiga E, Katamanywa J, Bamuhiiga J, Byamukama E, Agunyo S, Richards F. The disappearance of onchocerciasis from the Itwara focus, western Uganda after elimination of the vector Simulium neavei and 19 years of annual ivermectin treatments. Acta Trop. 2013; 126(3): 218–21. |
| 334422 | Uganda | Okello DO, Ovuga EB, Ogwal-Okeng JW. Dermatological problems of onchocerciasis in Nebbi District, Uganda. East Afr Med J. 1995; 72(5): 295–8. |


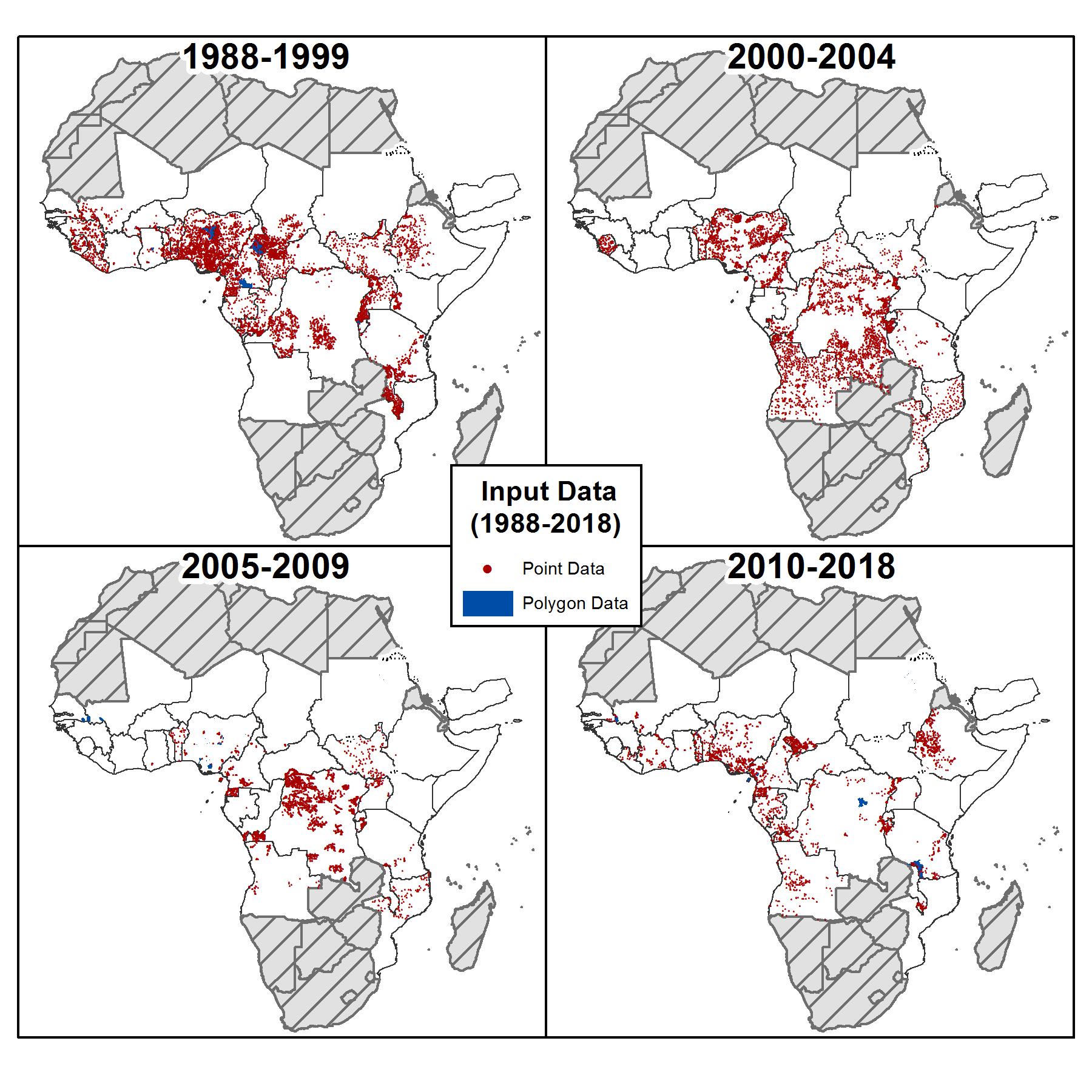


Supplementary Figure 3. Africa and Yemen data coverage maps.

Locations of the unique point and polygon data used in modelling, grouped by years: 1988–1999, 2000–2004, 2005–2009, and 2010–2018. Hatched countries were not included in the MBG modelling region. Data that were able to be matched to point coordinates are represented by red dots and data that represented a larger area are represented by blue polygons.

## **3.5 APOC survey year matching**

The African Programme for Onchocerciasis Control (APOC),(3) which operated from 1995 to 2015, relied on Rapid Epidemiological Mapping of Onchocerciasis (REMO) to map baseline onchocerciasis prevalence and endemicity in 19 onchocerciasis-endemic African countries outside the Onchocerciasis Control Programme (OCP) focal region (APOC was later expanded to additional countries). REMO(4,5) mapping, involving village-level surveys of nodule (onchocercoma) prevalence among adult males, was completed for 12 APOC countries by 2001.(6) We obtained these pre-2001 nodule mapping data, when available, from the ESPEN data portal, although detailed information on survey years were lacking for many (N = 4409) of these datapoints. So that these essential baseline mapping surveys could inform our spatiotemporal model of onchocerciasis prevalence, we investigated the pre-2001 REMO mapping surveys in APOC countries and attempted to narrow the possible range of survey years for these data. As widespread use of REMO by APOC began in 1996,(6) and these data were indicated by the ESPEN database as representing pre-2001 surveys, we considered 1996 and 2000 as the initial lower and upper bounds for survey years. We next identified survey locations for which the first year of onchocerciasis MDA (mass drug administration) occurred prior to 2001 (see section 4.2 below) and assumed that baseline mapping occurred at that location no later than the preceding year, treating that year as the new upper bound. Finally, we examined the available literature on pre-2001 APOC REMO surveys, by country, to further narrow the possible range of survey years. Our decisions around survey year estimates are summarised in Supplementary Table 4. For each datapoint, we specified the final survey year as the median of the lower and upper year bounds (rounding down), considering this approach to balance the potential conflicting biases from incorrect survey years on the temporal trends in the MBG model and the association with environmental and sociodemographic covariates in child models.

Survey year data were also missing for some Phase 1a and 1b monitoring and evaluation surveys in the APOC data retrieved from ESPEN. We identified survey years for most of these data by matching them to the locations, sample sizes, cases, and number of villages reported by Tekle et al.,(7) supplemented with additional sources(8,9) for some countries. We identified survey years for a total of 1001 observations. Survey years could not be reliably identified for some Phase 1a and 1b data, specifically Phase 1a data for Kasai Occidental (Democratic Republic of the Congo, DRC), Phase 1a and 1b data for Bioko Island (Equatorial Guinea), Phase 1a data for Kogi (Nigeria), and some Phase 1b data for Uganda; these data were excluded from our analysis. We assumed sample age ranges of 5–99 years of age for Phase 1a and 1b data(7) unless the ESPEN dataset indicated otherwise.

**Supplementary Table 4. APOC REMO survey year investigations.** CDTI: Community-directed treatment with ivermectin.

| **Country** | **Data Rows** | **Notes** | **Survey Year Strategy** |
| --- | --- | --- | --- |
| Central African Republic | 875 | CDTI began in some areas in 1993, following REMO surveys.(10) | Use 1992 (one year prior to CDTI initiation) as lower bound. |
| Cameroon | 389 | Early proof-of-concept tests of the REMO methodology were performed in Cameroon in 1993.(4) | Assume 1993 as lower bound. |
| Democratic Republic of Congo | 684 | REMO began in 1997 in certain foci and continued until 2008;(11) CDTI areas were established in 1999 based on REMO.(12) | REMO appears to have been undertaken between 1997 and 1999 (although Bof(11) noted that it continued through 2008; ESPEN data include surveys beyond 2001). Assume 1997 as lower bound for pre-2001 DRC nodule surveys. |
| Republic of Congo | 290 | No information found except an MDA start year of 2001 for a small subset of these locations. | Assume 1996 as lower bound. |
| Ethiopia | 283 | Country-wide REMO was performed in Ethiopia in 1997 and 2001.(13) | Use 1997 as survey year for pre-2001 data. |
| Kenya | 93 | No information found. | Assume 1996 as lower bound for pre-2001 data. |
| Nigeria | 576 | REMO was reported in 1995 to have been used for nationwide mapping;(3) another source indicated that REMO was carried out in Nigeria between 1994 and 1996.(14) | Assume 1994 and 1995 as lower and upper bounds of survey years for pre-2001 data. |
| Rwanda | 90 | Nationwide REMO was performed in 1999.(15) | Use 1999 as survey year for pre-2001 data. |
| Sudan | 2 | No information found. | Assume 1996 as lower bound. |
| South Sudan | 110 | REMO was performed between 1995 and 2002.(16) | Assume 1995 as lower bound. |
| Chad | 481 | REMO performed prior to 1999.(17) | Assume 1996 as lower bound, and 1998 as upper bound for surveys otherwise lacking upper bound estimates based on MDA. |
| Tanzania | 142 | Years of initiation of CDTI in Tanzania ranged from 1995 to 2004.(7) | Use 1994 as lower bound estimate. |
| Uganda | 394 | Nationwide REMO conducted between 1993 and 1997.(18) | Use 1993 and 1997 as lower and upper bounds, respectively. |

# **4.0 Supplementary covariates**

## **4.1 Pre-existing covariates considered for analysis**

A variety of environmental and sociodemographic variables were used to predict all-age onchocerciasis prevalence. Where available, the finest spatiotemporal resolution of gridded datasets was used. Geospatial covariate rasters were resampled to ~ 5 km in GeoTiff format, for consistent modelling. Where data coverage was inconsistent between our standard mask and the input data, either a local average or nearest neighbour method (depending on data type) was used to fill spatial data gaps. Data from the nearest year available were used if covariate coverage did not include all model years. Supplementary Table 5 contains a full list of covariates considered in our analysis.

## **4.2 Creation of MDA covariate**

Data used in the creation of the onchocerciasis MDA covariate were downloaded from the ESPEN Portal in April 2019.(19) The ESPEN data included the MDA start year and cumulative number of MDA rounds for each IU. ArcGIS software was then used to join the MDA data to the NTD (Neglected Tropical Disease) Implementation Unit Shapefile maintained by the Task Force for Global Health. A custom polygon was created for Sudan by referencing annual reports from The Carter Center. The MDA covariate value was set calculated as the cumulative number of rounds (defined as total number of rounds implemented) for the IU, and then converted to a raster for use in geospatial analysis. Data for lymphatic filariasis MDA were obtained from WHO(20) for the years during which LF (lymphatic filariasis) MDA was conducted, by IU, and joined onto the IU shapefile maintained by ESPEN. So that the effects of MDA for onchocerciasis and lymphatic filariasis were not double-counted in locations where both were indicated, a composite MDA covariate was produced which indicated the cumulative number of years for which either onchocerciasis or lymphatic filariasis MDA was indicated. This composite covariate was used in modelling.

## **4.3 Covariate reduction**

High collinearity among covariates may lead to unstable model coefficients and unreliable predictions.^37^ To reduce this problem, we derived a reduced covariate set using analysis of variance inflation factors (VIF).(22) Starting with the full list of covariates, we iteratively removed covariates with the highest VIF values until all remaining covariates had a VIF below 3.0. The reduced covariate set was used in fitting the MBG model and for spatiotemporal predictions. Supplementary Table 5 indicates the covariates that were retained in the final model, with representative plots provided for each covariate in Supplementary Figure 4. All variables were matched to the year of reported prevalence data, without any temporal lags (eg, temperature values for the year 2000 were joined to prevalence data for the year 2000).

**Supplementary Table 5. Covariates considered or retained for modelling, 1988–2018.**

All covariates considered for inclusion as predictors of onchocerciasis prevalence. Short names are provided for reference in later figures and tables. Covariates retained after VIF analysis are indicated with a (+).

| **Covariate** | **Short Name** | **Retained in Final Model** | **Years Available** | **Source** | **Reference** |
| --- | --- | --- | --- | --- | --- |
| Aridity | crutsard | - | 1988–2018 | Climatic Research Unit Time-Series (CRUTS) | Harris, I., Osborn, T.J., Jones, P. et al. Version 4 of the CRU TS monthly high-resolution gridded multivariate climate dataset. Sci Data 7, 109 (2020). <https://doi.org/10.1038/s41597-020-0453-3>  University of East Anglia. Climatic Research Unit TS v. 4.04 dataset. Available at: https://crudata.uea.ac.uk/cru/data/hrg/ |
| Average daily mean temperature | crutstmp | + | 1988–2018 | CRUTS | Harris, I., Osborn, T.J., Jones, P. et al. Version 4 of the CRU TS monthly high-resolution gridded multivariate climate dataset. Sci Data 7, 109 (2020). <https://doi.org/10.1038/s41597-020-0453-3>  University of East Anglia. Climatic Research Unit TS v. 4.04 dataset. Available at: https://crudata.uea.ac.uk/cru/data/hrg/ |
| Average daily minimum temperature | crutstmn | - | 1988–2018 | CRUTS | Harris, I., Osborn, T.J., Jones, P. et al. Version 4 of the CRU TS monthly high-resolution gridded multivariate climate dataset. Sci Data 7, 109 (2020). <https://doi.org/10.1038/s41597-020-0453-3>  University of East Anglia. Climatic Research Unit TS v. 4.04 dataset. Available at: https://crudata.uea.ac.uk/cru/data/hrg/ |
| Average daily maximum temperature | crutstmx | - | 1988–2018 | CRUTS | Harris, I., Osborn, T.J., Jones, P. et al. Version 4 of the CRU TS monthly high-resolution gridded multivariate climate dataset. Sci Data 7, 109 (2020). <https://doi.org/10.1038/s41597-020-0453-3>  University of East Anglia. Climatic Research Unit TS v. 4.04 dataset. Available at: https://crudata.uea.ac.uk/cru/data/hrg/ |
| Diurnal temperature range | crutsdtr | + | 1988–2018 | CRUTS | Harris, I., Osborn, T.J., Jones, P. et al. Version 4 of the CRU TS monthly high-resolution gridded multivariate climate dataset. Sci Data 7, 109 (2020). <https://doi.org/10.1038/s41597-020-0453-3>  University of East Anglia. Climatic Research Unit TS v. 4.04 dataset. Available at: https://crudata.uea.ac.uk/cru/data/hrg/ |
| Vapor pressure | crutsvap | - | 1988–2018 | CRUTS | Harris, I., Osborn, T.J., Jones, P. et al. Version 4 of the CRU TS monthly high-resolution gridded multivariate climate dataset. Sci Data 7, 109 (2020). <https://doi.org/10.1038/s41597-020-0453-3>  University of East Anglia. Climatic Research Unit TS v. 4.04 dataset. Available at: https://crudata.uea.ac.uk/cru/data/hrg/ |
| Wet day frequency | crutswet | + | 1988–2018 | CRUTS | Harris, I., Osborn, T.J., Jones, P. et al. Version 4 of the CRU TS monthly high-resolution gridded multivariate climate dataset. Sci Data 7, 109 (2020). <https://doi.org/10.1038/s41597-020-0453-3>  University of East Anglia. Climatic Research Unit TS v. 4.04 dataset. Available at: https://crudata.uea.ac.uk/cru/data/hrg/ |
| Distance to rivers >25 m wide | distrivers25m | + | Synoptic | Natural Earth Data (derived) | Andreadis KM, Schumann GJ-P, Pavelsky T. A simple global river bankfull width and depth database. Water Resources Research. 2013;49(10):7164–8. |
| River width (largest river within 5x5 km cell) | river_size | + | Synoptic | Natural Earth Data (derived) | Andreadis KM, Schumann GJ-P, Pavelsky T. A simple global river bankfull width and depth database. Water Resources Research. 2013;49(10):7164–8. |
| Elevation | elevation | - | Synoptic | NOAA/NCEI | Young, A. H., K. R. Knapp, A. Inamdar, W. B. Rossow, and W. Hankins, 2017: “The International Satellite Cloud Climatology Project, H-Series Climate Data Record Product”, Earth System Science Data, in preparation. |
| Enhanced Vegetation Index (EVI) | evi_v6 | + | 2000–2018 | MODIS | Huete, A., Justice, C. & van Leeuwen, W. MODIS vegetation index (MOD 13) algorithm theoretical basis document. (1999).  USGS & NASA. Vegetation indices 16-Day L3 global 500m MOD13A1 dataset. Available at: https://lpdaac.usgs.gov/dataset_discovery/modis/m odis_products_table/mod13a1. (Accessed: 25th July 2017)  Weiss, D. J. et al. An effective approach for gapfilling continental scale remotely sensed timeseries. Isprs J. Photogramm. Remote Sens. 98, 106–118 (2014).  C. Schaaf, Z. Wang. (2015). MCD43A1 MODIS/Terra+Aqua BRDF/Albedo Model Parameters Daily L3 Global - 500m V006. NASA EOSDIS Land Processes DAAC.  http://doi.org/10.5067/MODIS/MCD43A1.006 |
| Population | worldpop | + | 1988–2018 | WorldPop | Lloyd, C. T., Sorichetta, A. & Tatem, A. J. High resolution global gridded data for use in population studies. Sci. Data 4, sdata20171 (2017).  World Pop. Get data. Available at: <http://www.worldpop.org.uk/data/get_data/>. (Accessed: 25th July 2017) |
| Growing season length | growingseason | - | Synoptic | FAO | FAO. GAEZ - Global Agro-Ecological Zones data portal. Available at: http://www.fao.org/nr/gaez/about-data-portal/en/. (Accessed: 25th July 2017)  FAO. GAEZ - Global Agro-Ecological Zones users guide. (2012). |
| Tassled cap brightness | tcb_v6 | + | 2000–2017 | MODIS | USGS & NASA. Nadir BRDF- Adjusted Reflectance Reflectance 16-Day L3 Global 1km dataset. Available at: https://lpdaac.usgs.gov/dataset_discovery/modis/m odis_products_table/mcd43b4. (Accessed: 25th July 2017)  Strahler, A. H. & Muller, J.-P. MODIS BRDF/Albedo product: algorithm theoretical basis document version 5.0. (1999).  Weiss, D. J. et al. An effective approach for gapfilling continental scale remotely sensed timeseries. Isprs J. Photogramm. Remote Sens. 98, 106–118 (2014).  C. Schaaf, Z. Wang. (2015). MCD43A1 MODIS/Terra+Aqua BRDF/Albedo Model Parameters Daily L3 Global - 500m V006. NASA EOSDIS Land Processes DAAC.  [http://doi.org/10.5067/MODIS/MCD43A1.006](https://mail02.ndc.nasa.gov/owa/redir.aspx?C=d8iOg4QbPKWhKvzStkXuvGFlmSiBWYwZ2UyoRRtvonwsmuff8aTTCA..&URL=http%3a%2f%2fdoi.org%2f10.5067%2fMODIS%2fMCD43A1.006) |
| Precipitation (Multi-source Weighted-Ensemble) | mswep | + | 1988–2016 | Princeton Climate Analytics | Beck, H.E., A.I.J.M. van Dijk, V. Levizzani, J. Schellekens, D.G. Miralles, B. Martens, A. de Roo: MSWEP: 3-hourly 0.25 global gridded precipitation (1979-2015) by merging gauge, satellite, and reanalysis data, Hydrology and Earth System Sciences, 21(1), 589-615, 2017. |
| Slope for land surfaces | slope | + | Synoptic | NOAA/NCEI | Young, A. H., K. R. Knapp, A. Inamdar, W. B. Rossow, and W. Hankins, 2017: “The International Satellite Cloud Climatology Project, H-Series Climate Data Record Product”, Earth System Science Data, in preparation. |
| Soil: Saturated water content (200 cm depth) | sgawcts | - | Synoptic | SoilGrid | Hengl T, Mendesde Jesus J, MacMillan RA, Batjes NH, Heuvelink GBM, Ribeiro E, Samuel-Rosa A, Kempen B, Leenaars JGB, Walsh MG, Gonzalez MR. “SoilGrids1km — Global soil information based on automated mapping.” *PLOS ONE* 9(8): e105992. 29 Aug 2014. doi:10.1371/journal.pone.0105992 |
| Soil: Probability of bedrock exposure | sgbdrlog | + | Synoptic | SoilGrid | Hengl T, Mendesde Jesus J, MacMillan RA, Batjes NH, Heuvelink GBM, Ribeiro E, Samuel-Rosa A, Kempen B, Leenaars JGB, Walsh MG, Gonzalez MR. “SoilGrids1km — Global soil information based on automated mapping.” *PLOS ONE* 9(8): e105992. 29 Aug 2014. doi:10.1371/journal.pone.0105992 |
| Soil: Bulk density (200 cm depth) | sgbldfie | + | Synoptic | SoilGrid | Hengl T, Mendesde Jesus J, MacMillan RA, Batjes NH, Heuvelink GBM, Ribeiro E, Samuel-Rosa A, Kempen B, Leenaars JGB, Walsh MG, Gonzalez MR. “SoilGrids1km — Global soil information based on automated mapping.” *PLOS ONE* 9(8): e105992. 29 Aug 2014. doi:10.1371/journal.pone.0105992 |
| Soil: Clay content mass fraction (200 cm depth) | sgclyppt | + | Synoptic | SoilGrid | Hengl T, Mendesde Jesus J, MacMillan RA, Batjes NH, Heuvelink GBM, Ribeiro E, Samuel-Rosa A, Kempen B, Leenaars JGB, Walsh MG, Gonzalez MR. “SoilGrids1km — Global soil information based on automated mapping.” *PLOS ONE* 9(8): e105992. 29 Aug 2014. doi:10.1371/journal.pone.0105992 |
| Soil: Coarse fragments (200 cm depth) | sgcrfvol | + | Synoptic | SoilGrid | Hengl T, Mendesde Jesus J, MacMillan RA, Batjes NH, Heuvelink GBM, Ribeiro E, Samuel-Rosa A, Kempen B, Leenaars JGB, Walsh MG, Gonzalez MR. “SoilGrids1km — Global soil information based on automated mapping.” *PLOS ONE* 9(8): e105992. 29 Aug 2014. doi:10.1371/journal.pone.0105992 |
| Cumulative years of mass drug administration (MDA) for lymphatic filariasis or onchocerciasis | allmda | + | 1988–2018 | WHO (rasters produced in current study) | WHO. Expanded Special Project for Elimination of Neglected Tropical Diseases (ESPEN). https://www.espen.org/ (accessed May 20, 2020).  WHO. Global Programme to Eliminate Lymphatic Filariasis. http://www.who.int/lymphatic_filariasis/elimination- programme/en/ (accessed May 29, 2019). |


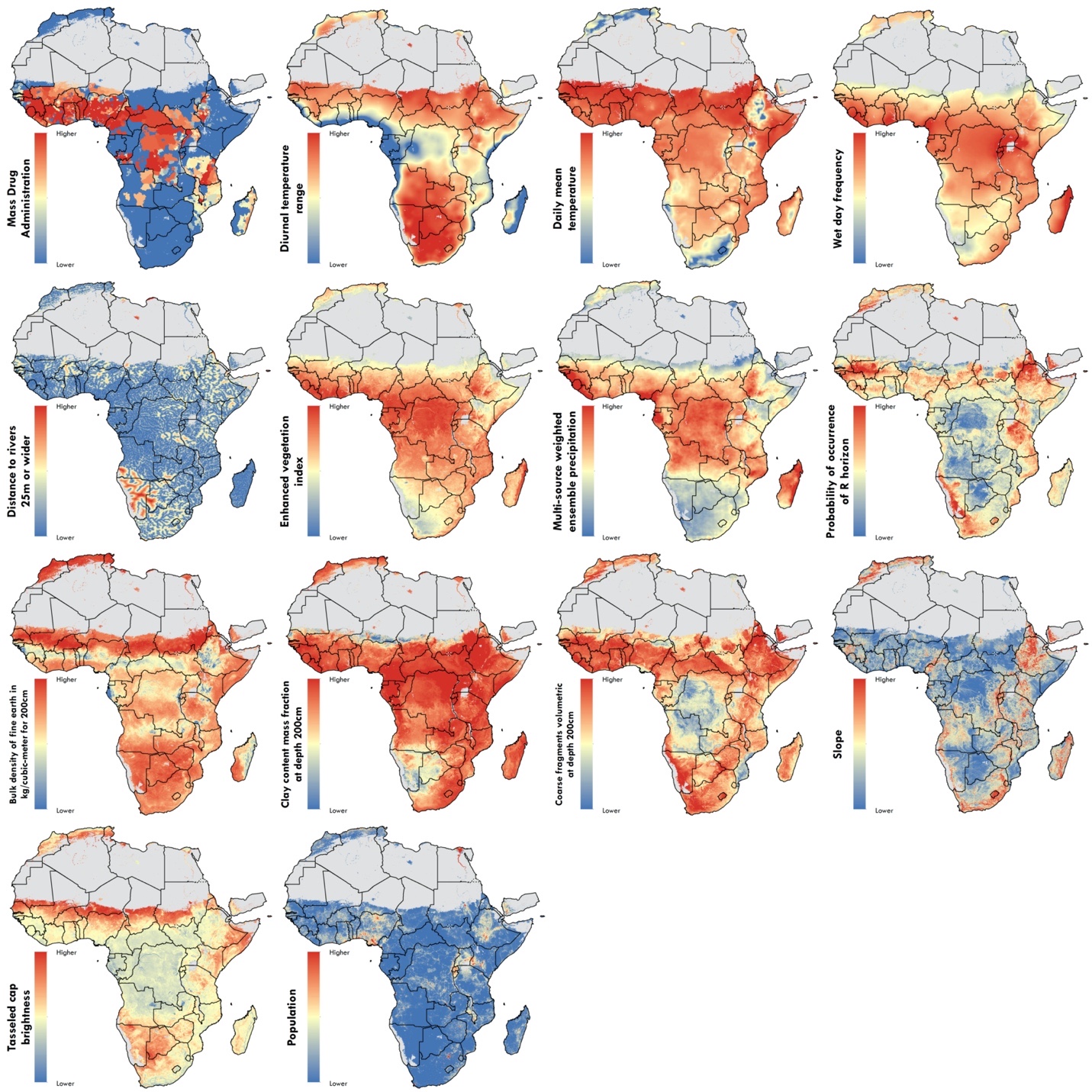


Supplementary Figure 4. Africa and Yemen covariate values.

15 environmental or sociodemographic variables were used as inputs for the modelling process for Africa and Yemen (river size is not shown in this figure). Time-varying covariates are presented here for the year 2015. Please refer to Supplementary Table 5 for the corresponding citations for each covariate.

# **5.0 Supplementary methods**

## **5.1 Age and diagnostic crosswalks**

Surveys of onchocerciasis prevalence have varied in both their sampled age ranges and in the diagnostic tests they utilised. For example, epidemiological surveys during the OCP program relied on skin snip (microfiladermia) examinations of individuals aged 5 years or older,(23,24) while APOC relied on the REMO (Rapid Epidemiological Mapping of Onchocerciasis)(5,6,25,26) methodology to identify endemic areas requiring MDA, employing nodule palpation in adults. To harmonise these data sources, we adjusted survey data to represent all-age microfiladermia prevalence by developing and applying an age and diagnostic crosswalk model that simultaneously accounts for typical age-dependent trends in prevalence and differential detection sensitivity between skin snip examination and nodule palpation. We did not crosswalk results from antibody (eg, Ov16) surveys as these data were not used in fitting the MBG model.

We identified published within-study comparisons reporting results from skin snip biopsies (microfiladermia), nodule (onchocercoma) palpation, or both, for more than one age group in a given study population. Study cohorts with zero reported cases were excluded from the crosswalk training dataset because they do not contribute information with which to derive age trends in endemic settings. Studies were eligible for inclusion in the crosswalk training dataset if they reported data from a country in our MBG modelling region; all eligible studies reported data from surveys conducted in our geospatial modelling timeframe (1988–2018). We identified 133 unique survey populations from 36 studies reporting skin snip prevalence in multiple age groups, and 126 unique survey populations from 22 studies reporting nodule prevalence in multiple age groups; among these surveys, a total of 100 unique survey populations from 19 studies reported both skin snip and nodule prevalence from the same study populations and age groups. Supplementary Table 6 summarises these sources.

**Supplementary Table 6. Data used in estimation of age and diagnostic crosswalk.**

Note that studies may have reported data for additional diagnostic tests that were not used in crosswalk model fitting.

| **Countries** | **Survey Years** | **Diagnostic Data** |
| --- | --- | --- |
|  |  |  |
| Benin(27) | 1991 | skin snip, nodule |
| Cameroon(28) | 1992 | skin snip |
| Cameroon(29) | 1992 | skin snip, nodule |
| Cameroon(30) | 1996, 2005, 2006, 2011 | skin snip, nodule |
| Cameroon(31) | 2000 | nodule |
| Cameroon(32) | 2008 | skin snip, nodule |
| Cameroon(33) | 2009 | skin snip, nodule |
| Cameroon(34) | 2009 | skin snip, nodule |
| Cameroon(35) | 2012 | skin snip, nodule |
| Cameroon(36) | 2015 | skin snip, nodule |
| Cameroon(37) | 2015 | skin snip, nodule |
| Cameroon(38) | 2015 | skin snip, nodule |
| Cameroon, Uganda(39) | 1993 (Uganda), 1996 (Cameroon), 2005 (both countries) | skin snip, nodule |
| Central African Republic(40) | 1990, 1995 | skin snip |
| Ethiopia(41) | 1994 | skin snip, nodule |
| Ethiopia(42) | 1997 | skin snip |
| Ethiopia(43) | 2005 | skin snip |
| Ethiopia(44) | 2006 | skin snip |
| Ethiopia(45) | 2012 | skin snip |
| Gabon(46) | 2004 | nodule |
| Nigeria(47) | 1992 | skin snip |
| Nigeria(48) | 1994 | skin snip, nodule |
| Nigeria(49) | 1994 | skin snip |
| Nigeria(50) | 1997 | skin snip |
| Nigeria(51) | 2000 | skin snip, nodule |
| Nigeria(52) | 2005 | skin snip |
| Nigeria(53) | 2007 | skin snip |
| Nigeria(54) | 2008 | skin snip, nodule |
| Nigeria(55) | 2008 | skin snip |
| Nigeria(56) | 2008 | skin snip, nodule |
| Nigeria(57) | 2009 | skin snip |
| Nigeria(58) | 2009 | skin snip, nodule |
| Nigeria(59) | 2010 | nodule |
| Sierra Leone (60) | 2010 | skin snip |
| Togo(61) | 1992 | skin snip |
| Togo(62) | 2014 | skin snip |
| Togo(63) | 2015 | skin snip |
| Uganda(64) | 1993 | skin snip, nodule |
| Uganda(65) | 2012 | skin snip, nodule |

We first retrieved population estimates by single age-year from the Global Burden of Disease (GBD)(66) for the country and year of each survey in the crosswalk training set. We assumed that the age distribution ($P\left( A \right)$, or probability of age $A$) within a survey sample matched that in the country and year of the survey, and estimated prevalence-by-age models ($P\left( D|A \right)$, or the probability of disease, $D$, at age $A$) from birth through age 94 years, the maximum individual age-year modelled by GBD. We used the GBD age distributions to estimate the likelihood of observing the reported number of cases in each surveyed age bin, given a logistic (binomial) regression model of the average prevalence-by-age relationship across surveys:

$$\mathrm{logit}\left( P\left( D | A \right) \right)= \beta_{0}+I_{\mathrm{ss}}f_{\mathrm{ss}}\left( A \right)+ I_{\mathrm{nod}}f_{\mathrm{nod}}\left( A \right)+\alpha_{i}+{I_{\mathrm{ss}}\beta}_{\mathrm{ss}}$$

Onchocerciasis prevalence at a given age ($P\left( D | A \right)$) was modelled in logit space as a linear combination of an intercept, $\beta_{0}$, which was set at logit(0.00001) to drive prevalence at birth toward zero; basis splines (fda R package(67)) on age, $f\left( A \right)$, to accommodate non-linear age trends; $\alpha_{i}$, cohort-level fixed effects (indicator variables identifying study cohorts in the training dataset) to account for differences in study populations and survey designs; and a fixed diagnostic effect for skin snip surveys, $\beta_{\mathrm{ss}}$, to model overall differences between skin snip and nodule surveys. Diagnostic indicator variables, $I_{\mathrm{ss}}$ and $I_{\mathrm{nod}}$, were set to 1 for skin snip and nodule observations, respectively, and set to 0 otherwise, to select the appropriate age and diagnostic effects for a given survey. Study cohort effects were assumed to be shared across diagnostic tests. The likelihood function was evaluated as the sum, across studies (*i*) and age bins ($A1\leq A<A2$), of the binomial likelihood of observing the reported numbers of cases given the reported sample sizes, age ranges, and diagnostic tests, using population-weighted mean prevalence values evaluated at the midpoints of each age year.

Spline knot placements were identified by spacing four internal knots evenly by quantile in the training data, determined separately for skin snip and nodule surveys, with additional knots placed at ages 3, 6, and 65 to help stabilise model behaviour in early childhood and older adulthood. Starting values for all spline, cohort and diagnostic coefficients were randomly drawn from uniform distributions in the interval [–5, 5]. Models were estimated with maximum likelihood optimisation using the box constraint quasi-Newton algorithm^66^ (without constraints) in the optim function (R stats package(69)).

Prior to running the MBG model, all survey data that derived from a restricted age range (ie, anything other than 0–94 years), or that represented nodule prevalence, were adjusted using the fitted crosswalk model. For each individual survey population, the crosswalk model was refit via maximum likelihood to the prevalence data for that survey, with all coefficients other than the cohort-level effect fixed, $\alpha_{i}$, fixed to their estimates from the full crosswalk model. This newly estimated value of $\alpha_{i}$ was then re-inserted into the crosswalk model with the other coefficients fixed, to calculate all-age skin snip prevalence for that survey sample. Crosswalked estimates were then used as the outcome measure in the MBG model.

Surveys that reported prevalence of 0% or 100% were particularly difficult to interpret for crosswalk purposes. A report of 0% prevalence could reflect true absence of infection, insufficient diagnostic sensitivity, or sampling variance in small samples, while a report of 100% prevalence masks any implicit linkage between prevalence and the scale of infection intensity. While many of these concerns also apply to surveys reporting intermediate prevalence, the particular uncertainty involved with surveys reporting 0% or 100% prevalence increases the risk of inappropriate crosswalking, and for this reason we did not crosswalk such surveys.

Uncertainty in the crosswalk models was estimated using bootstrap analysis, with 100 replicates generated by sampling, with replacement, an equal number of study cohorts as in the full crosswalk training dataset. Resampling was conducted by cohort rather than age bin to better estimate variation among surveys. Spline knot locations and model coefficients were estimated for each bootstrap replicate independently of other replicates and of the full model. To visualise bootstrapped results for a given survey, cohort effects were calculated for each replicate using maximum likelihood optimisation with all other coefficients fixed to the estimated values for the replicate.


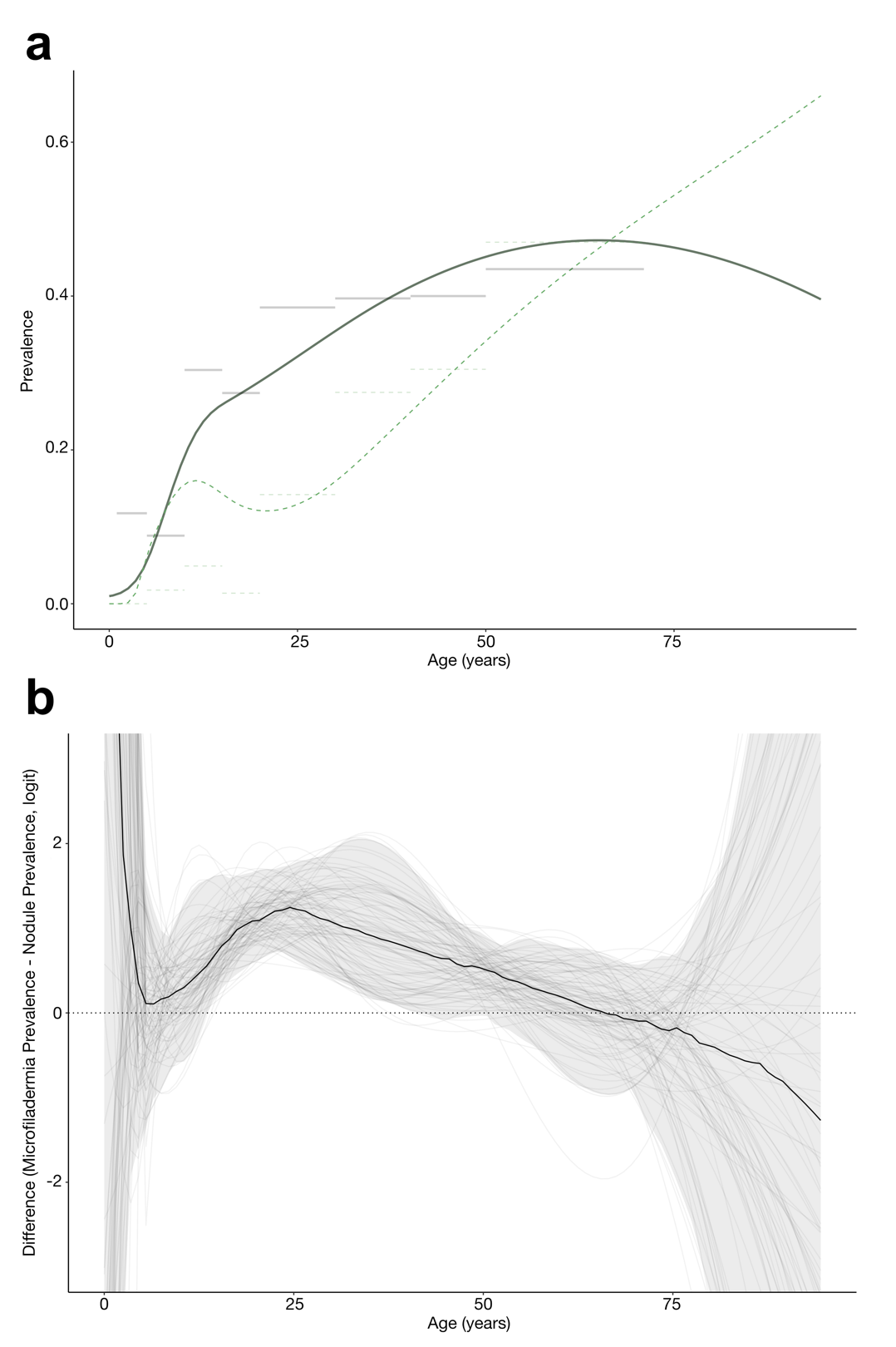


Supplementary Figure 5. Diagnostic and age crosswalk model.

Outputs from the diagnostic and age crosswalk model. (**a**) Example prevalence-by-age curves fit to a study from Benin(27) that reported both skin snip and nodule age-binned data and was included in the crosswalk model training dataset. Estimates are shown for skin snip microfiladermia prevalence (solid black line) and nodule prevalence (dotted green line); horizontal bars indicate the age-binned prevalence data reported by that study (solid gray: skin snip; dotted green: nodule). **(b)** Bootstraped estimates of differences between microfiladermia (skin snip) and nodule prevalence by age, in logit space. Each of 100 bootstrap samples is shown (faint grey line) along with the median estimate (solid black line) and central 95% uncertainty interval (shaded area). Positive values indicate higher skin snip prevalence than nodule prevalence.

In an example location with moderately high microfiladermia prevalence, our final crosswalk model (Supplementary Figure 5) suggests a sharp increase in prevalence through childhood and adolescence, with a slower increase between approximately ages 15 and 65 years. This general pattern is qualitatively consistent with the range of onchocerciasis microfiladermia age trends reported by other studies,(70–72) although substantial site-specific variation exists. The relationship between microfiladermia and nodule prevalence within onchocerciasis-endemic communities was previously modelled by Coffeng et al.,(73) using pre-control data from a broad sampling of sites in OCP and APOC regions. Their multivariate logistic regression model, relating nodule prevalence in adult males (aged 20 years or older) to microfiladermia prevalence in individuals aged 5 years or older, did not explicitly model changes in prevalence with age, but revealed higher prevalence estimates from skin snip biopsies than those from nodule palpation surveys in the same communities. Our full crosswalk model and bootstrapped uncertainty estimates (Supplementary Figure 5) are qualitatively consistent with their results, estimating that skin snip prevalence exceeds nodule prevalence, on average, from adolescence until about age 50, with poorer resolution of this relationship in children and older adults.

While crosswalking survey data that are based on differing diagnostics and age coverage enabled us to leverage a more comprehensive geospatial dataset than is otherwise tractable, we identify several limitations to our crosswalk approach. Our model assumes that, for a given diagnostic approach, changes in prevalence by age follow a consistent pattern across locations, years, sexes, and programmatic contexts. However, actual and reported microfiladermia and nodule prevalence patterns are influenced by local factors including ecological conditions; infection intensity; vector identity, density, exposure, and control history; MDA coverage; variations in survey sampling designs and in diagnostic specificity and sensitivity; and sex.(70,72–79) We have not modelled the age- and diagnostic-specific effects of MDA on prevalence in the crosswalk models (although MDA is included as a predictor during the MBG modelling stage). We were also limited by the variable reporting of age information among data sources. Some surveys did not report age ranges, and for these surveys we assumed that their data represented all-age prevalence, risking possible misclassification. The absence of individual-level data on onchocerciasis prevalence also precluded full age-standardisation, as we could only assume that the survey sample matched the age structure of the general population. Our crosswalks do not currently account for the sensitivity and specificity of skin snip and nodule diagnostics, and crosswalk uncertainty is therefore likely to be underestimated. We also do not currently have a computationally feasible method to propagate uncertainty from the crosswalk models into the MBG models in a way that accounts for the inconsistent reporting of sampled age ranges among studies.

## **5.2 Polygon resampling**

Prevalence records are representative of either georeferenced point locations or polygonal areas (eg, as defined by the borders of administrative or programmatic units). As our modelling framework relies on coordinate-referenced data in order to fit the continuous spatial random fields, we converted areal data to a representative collection of point data. This “polygon resampling” process, described previously for geostpatial modelling of under-5 mortality,(80) generates candidate locations based on the underlying population density of the resampled area, implicitly assuming that surveys employed population-based designs, and is illustrated in Supplementary Figure 6 using Cameroon in 2015 as an example. For each polygonal observation in our dataset, 10 000 points were randomly sampled from within the polygon, with weighting by the WorldPop total population raster. Candidate points were clustered using k-means clustering, generating a set of final points with a density of 1 per 1000 grid cells, except for small polygons, in which case density was iteratively increased by a factor of 10 until a minimum threshold of 10 points was achieved. Weights were assigned to each point proportionally to the number of candidate points that entered into the k-means cluster. The points generated by this resampling process were assigned the prevalence of onchocerciasis reported for the survey for that polygon. These sample weights were used in MBG model fitting within INLA.


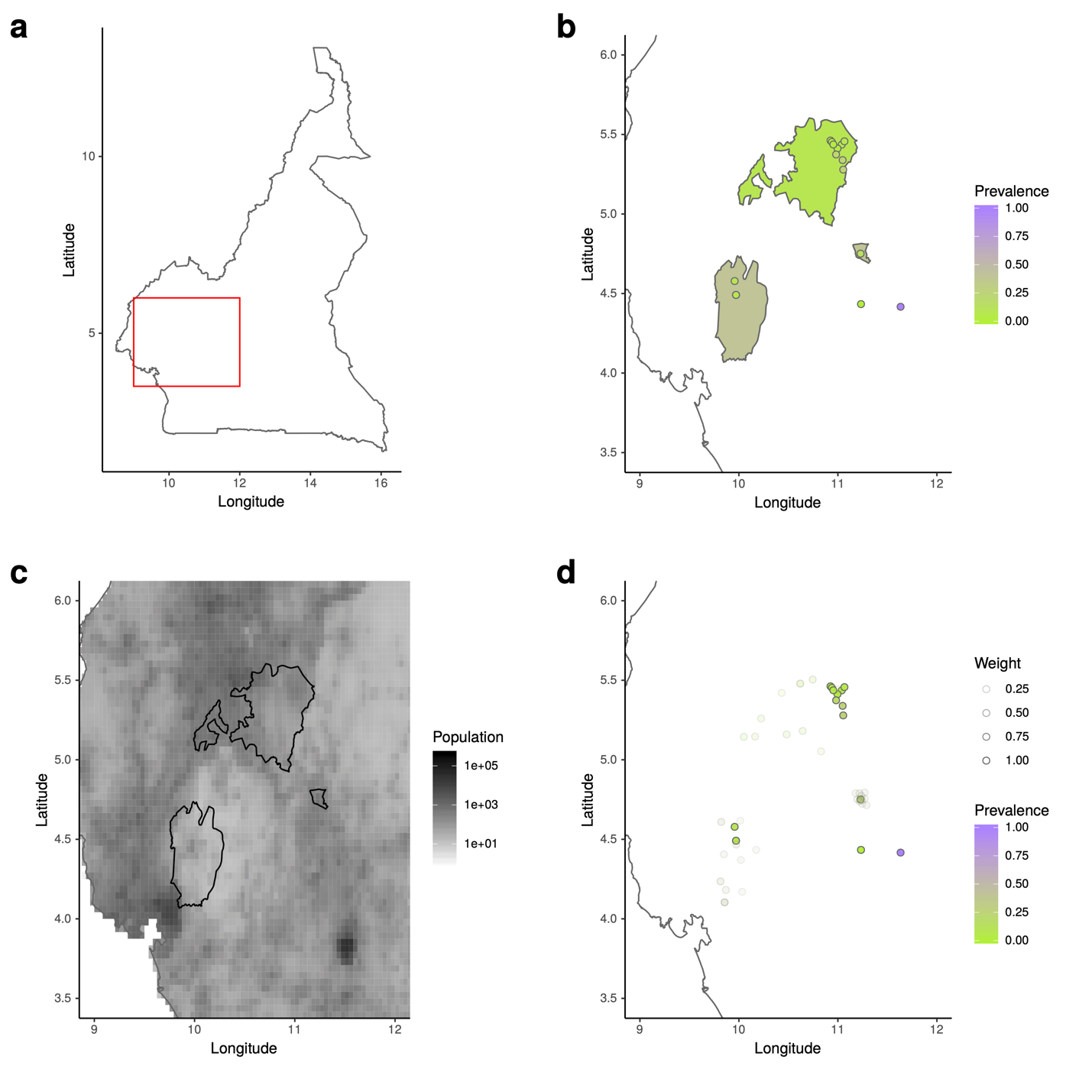


Supplementary Figure 6. Polygon resampling.

The process of polygon resampling is illustrated using reported onchocerciasis prevalence data for Cameroon in 2015. (**a**) National map of Cameroon, showing the inset area (red frame) that is featured in the remaining panels. (**b**) Reported areal (irregular polygons) and point-level (circles) prevalence data. (**c**) Underlying population surface from WorldPop (displayed on a log_10_ scale), with survey polygons overlayed. Polygons are resampled proportionally to 5 x 5 km population density. (**d**) Locations of the final datapoints for geospatial modelling, showing both those data originally reported with coordinates and those derived by resampling the polygon data. The opacity of resampled datapoints in (d) reflects their relative weights, which sum to 1.0 within an individual areal survey.

## **5.3 Geostatistical model**

### 5.3.1 Model geographies and time period

Model-based geostatistical (MBG) methods were used to generate estimates of all-age onchocerciasis microfiladermia prevalence for onchocerciasis-endemic countries of Africa, plus Yemen (listed in Supplementary Table 2). A single model was fit to this geographical region. We did not model countries in the Western Hemisphere that were formerly or are currently endemic for onchocerciasis, due to their highly localised onchocerciasis foci. We were principally concerned with estimates for the time period 2000–2018, but fit the model using data from 1988–2018 in order to leverage information from pre-2000 OCP and APOC surveys, and thereby improve “baseline” (ie, year 2000) model estimates in countries covered by those programmes. Reporting of results in the main text focuses on estimates for 2000–2018.

### 5.3.2 Covariate coverage

As with any regression model, the reliability of predictions from our model is affected by the overlap between covariate values in training and prediction datasets. Predictions in regions with a range of covariate values that fall outside the range of values in the training set may be prone to extrapolation errors. Supplementary Figure 7 illustrates the number of covariates whose mean values (averaged over 1988–2018) fall outside the central 95% quantile interval of values in the training set, for each 5 x 5-km pixel. Child model predictions in areas with poor covariate representation (ie, a large number of covariates with values outside the central interval) should be considered with special caution. These areas include the Sahel and Sahara, as well as Yemen, Kenya, Somalia, eastern Ethiopia, and southern Angola.

### 5.3.3 Environmental suitability

Cromwell et al. recently produced an environmental suitability model for onchocerciasis, using a boosted regression tree (BRT) model trained with space- and time-referenced data on onchocerciasis occurrence. (81) In contrast to our present MBG model, the Cromwell suitability model leveraged data from not only skin snip and nodule palpation surveys, but also Ov16 serosurveys, onchocerciasis-derived eye or skin morbidity surveys, and other diagnostics. The model was trained using an overlapping but non-identical set of environmental covariates as those used in the present study, including climatic, topographic, hydrologic, vegetative, and urbanicity variables. Pseudo-absence records were generated using background sampling. Importantly for our present purposes, this suitability model does not incorporate the influence of programmatic interventions for onchocerciasis and does not use data on onchocerciasis absence, providing a reflection of *O. volvulus* endemicity apart from temporal changes in infection prevalence. The outputs of the BRT model represent a spatially explicit index from 0–1, reflecting environmental suitability for onchocerciasis occurrence in a given 5 x 5-km cell. We incorporated mean predictions from this suitability model, using covariate values for 2016 (Supplementary Figure 8), to improve the behaviour of our MBG model in areas of poor prevalence data coverage, particularly in areas where covariates lie outside the range of values in sampled locations and MBG predictions are therefore subject to extrapolation uncertainty.

**
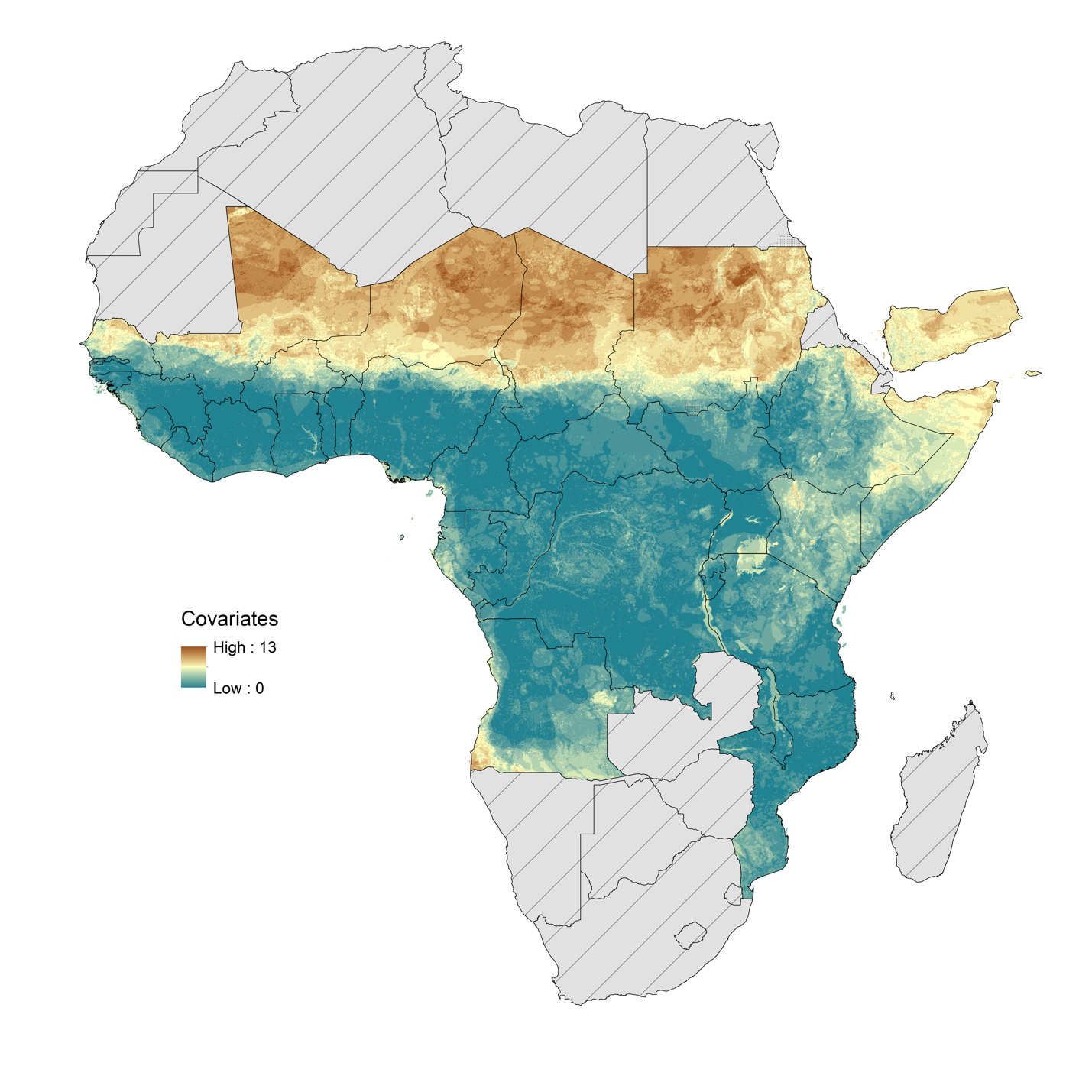
**

Supplementary Figure 7. Number of covariates with values outside the central 95% interval of values at survey sites.

For each 5 x 5-km pixel, the number of covariates (out of a total of 16) whose average value lies outside the central 95% interval of values at observed survey sites is indicated. Localities with a large number of such covariates represent areas in which predictions are less reliable, due to potential extrapolation error.


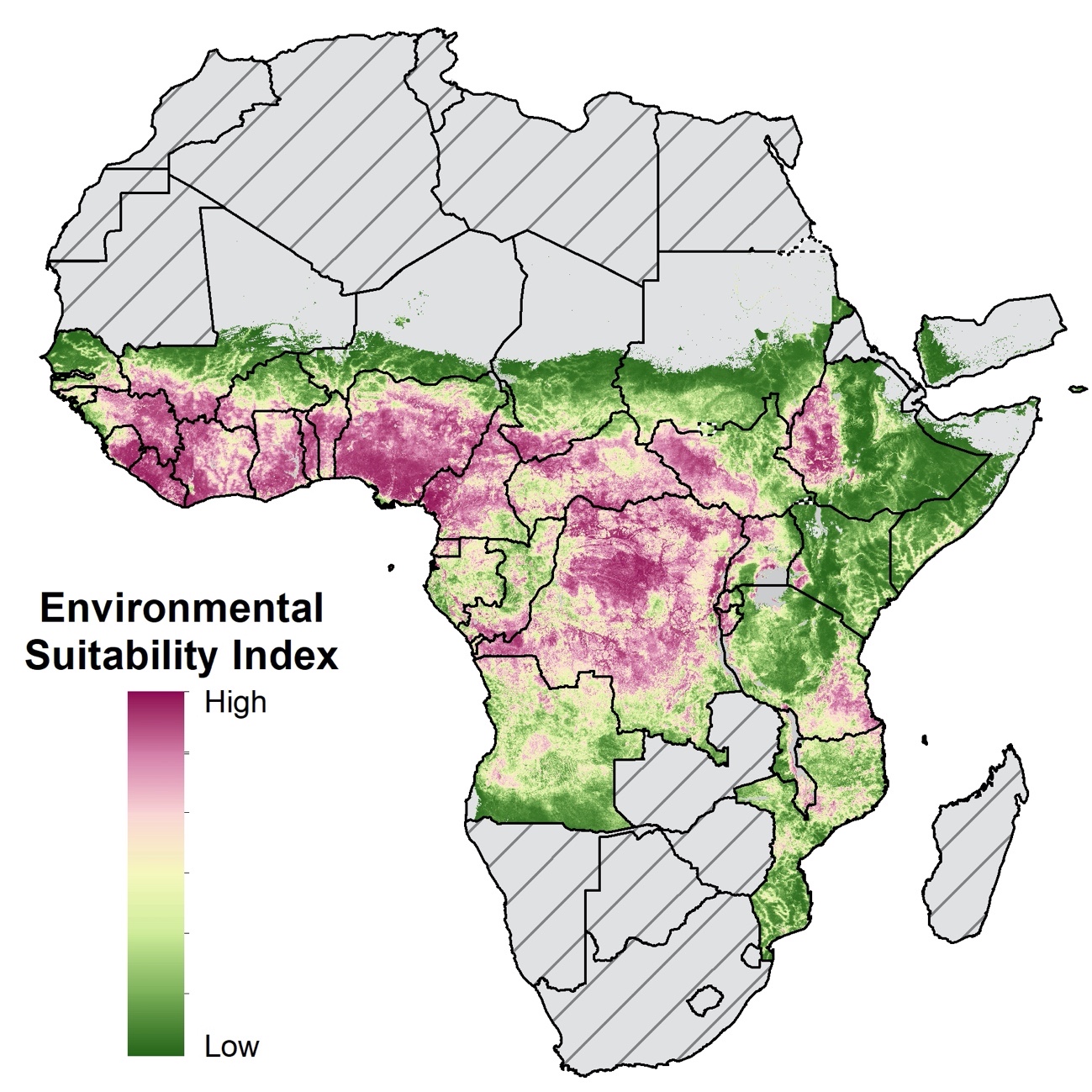


Supplementary Figure 8. Onchocerciasis suitability model predictions (Cromwell et al., 2021) for 2016.

Mean environmental suitability for onchocerciasis, from a previous boosted regression tree (BRT) analysis (81) of onchocerciasis presence data, using covariate values for 2016.

### 5.3.4 Model description

We modelled *Onchocerca volvulus* infection prevalence using a spatially explicit Bayesian generalised linear mixed effects regression model in R-INLA:

$$Y_{i,t}\sim Binomial\left( p_{i,t}, N_{i,t} \right)$$

$$\mathrm{logit}\left( p_{i,t} \right)= \beta_{0}+ \beta X_{i,t} {+ \gamma}_{c\left[ i \right]}+U_{i} {+ Z}_{i}+ \epsilon_{i,t}$$

$$Z_{i}\sim GP(0, \Sigma_{space})$$

$$\epsilon_{i,t}\sim N\left( 0, \sigma_{nug}^{2} \right)$$

The number of infected individuals ($Y_{i,t}$) among a sample ($N_{i,t}$) in location *i* and year *t* was modelled as a binomial variable. This model specifies logit-transformed infection prevalence ($p_{i,t}$) as a linear combination of an intercept for the modelling region ($\beta_{0}$); covariate fixed effects (coefficients, $\beta$, and values, $X_{i,t}$); country random effects ($\gamma_{c\left[ i \right]}$); a second-order random walk model on estimates of onchocerciasis environmental suitability ($U_{i}$); a spatially correlated random field ($Z_{i}$); and an uncorrelated error term or nugget effect ($\epsilon_{i,t}$). The spatial random field ($Z_{i}$) was modelled as a Gaussian process with mean 0 and a Matérn covariance function ($\Sigma_{space}$). Modelled outputs from the BRT analysis of onchocerciasis environmental suitability ($U_{i}$), described in section 5.3.3, were modelled with a second-order random walk, with values grouped into 25 bins by quantile. This random walk model accommodates non-linearity in the relationship between suitability (based on presence-absence data) and the linear predictor. The INLA (integrated nested Laplace approximation) model was fit using an “empirical Bayes” integration strategy, which relies on mode values to approximate hyperparameter posterior distributions during estimation and enabled us to achieve tractable model computation times.(82)

### 5.3.5 Priors

We specified minimally informative priors for INLA hyperparameters, as detailed in Supplementary Table 7. Priors for the spatial hyperparameters $\tau$ and $\kappa$ were derived automatically by R-INLA based on the finite elements mesh.

**Supplementary Table 7. INLA model priors.**

| **Parameter** | **Description** | **Prior** |
| --- | --- | --- |
| $\beta_{0}$,$\beta$ | Intercept, covariate fixed effects | $N(\mu=0, \sigma^{2}=3^{2})$ |
| $\left( \frac{1}{\sigma_{country}^{2}} \right)$ | Precision for country random effects ($\gamma_{c\left[ i \right]},$i.i.d.) | $\mathrm{gamma}\left( a=1, b=0.00005 \right)$ |
| $\left( \frac{1}{\sigma_{nug}^{2}} \right)$ | Precision for nugget effect ($\epsilon_{i,t},$ i.i.d) | $pc.prec\left( u=0.5, \alpha=0.05 \right)$^1^ |
| $\left( \frac{1}{\sigma_{U}^{2}} \right)$ | Precision for suitability model ($U_{i},$ RW2 model) | $\mathrm{gamma}\left( a=1, b=0.00005 \right)$ |
| $\sigma_{SPDE}$ | Standard error for SPDE model | $\mathrm{pc}\left( 3.0, 0.05 \right)$^2^ |
| Range | Range for SPDE model | $\mathrm{pc}\left( 0.06171913, 0.05 \right)$^3^ |
| ^1^PC prior for precision.  ^2^PC prior for SPDE σ, indicating a 5% probability that σ is greater than 3.  ^3^PC prior for SPDE range, indicating a 5% probability that range is less than 0.06161412 (5% of the maximum extent of the spatial mesh). | | |

### 5.3.6 Mesh construction

We modelled continuous spatial random effects using stochastic partial differential equations (SPDE) representations of Gaussian-Markov random field (GMRF) approximations of a spatially autocorrelated Gaussian process, using triangular finite element meshes as implemented in the R-INLA R package.(83–85) Due to the large geographical size of the model region, a spherical (S2) mesh was constructed in order to minimise distance distortions. Minimum and maximum edge lengths were set to 25 and 500 km, respectively, and a 1000-km external buffer was used to avoid artifacts at the edges of the spatial domain.(84) These values were chosen to yield denser mesh vertices in data-rich areas while maximising the overall spatial field resolution, within computational constraints. The spatial mesh is illustrated in Supplementary Figure 9.


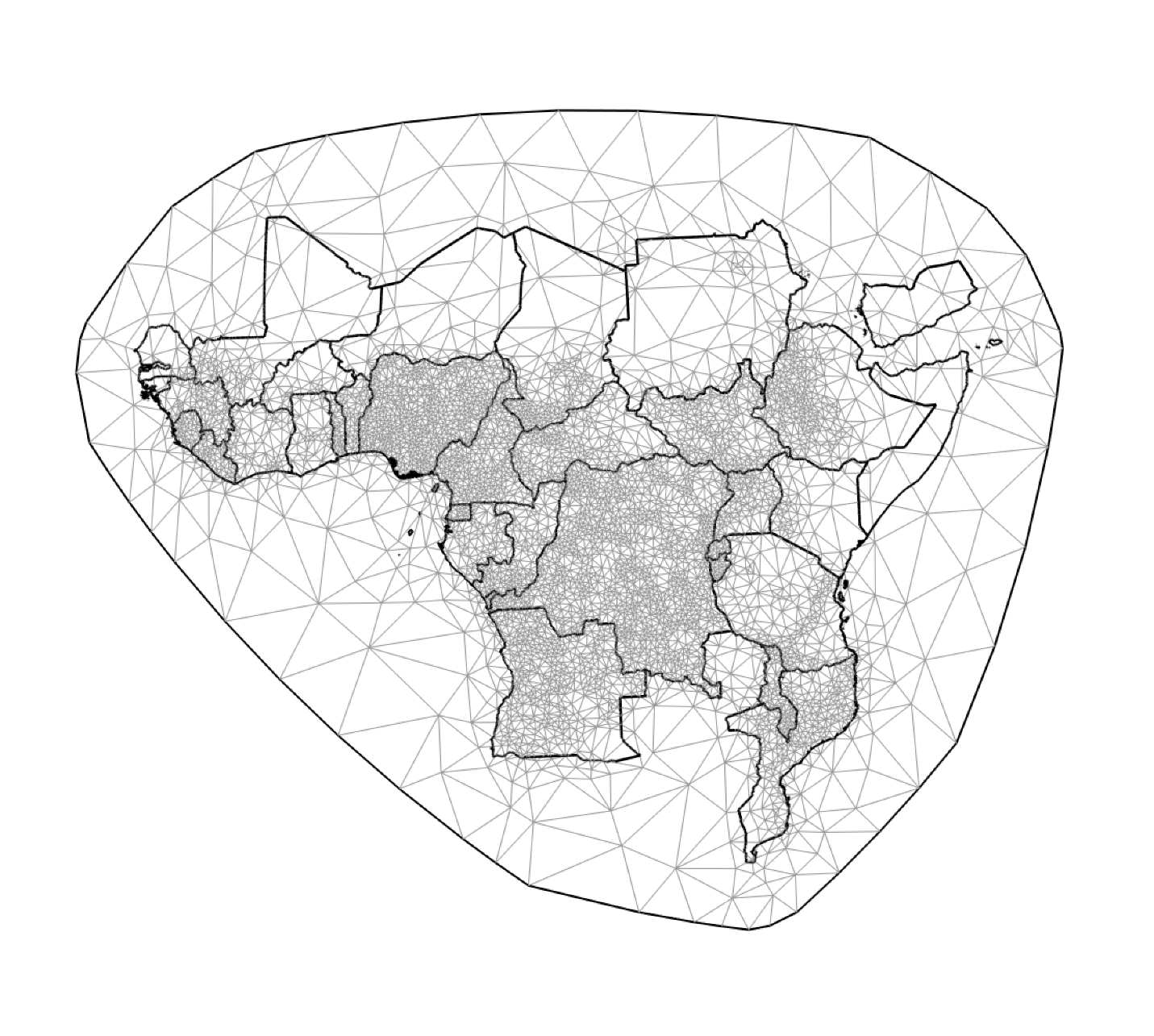


Supplementary Figure 9. Spatial mesh construction.

Two-dimensional projection of spherical refined Delaunay triangulation mesh used in estimating spatial random fields in Africa and Yemen, with national boundaries (bold lines). The mesh features greater vertex density in data-rich locations.

### 5.3.7 Model fitting and estimation generation

Models were fit using the integrated nested Laplace approximation (INLA) algorithm in R-INLA. Fitted models for each region were used to generate 1000 random samples from the joint posterior distributions of model parameters, yielding mean and uncertainty estimates for onchocerciasis prevalence.

### 5.3.8 Model results

Model parameter estimates from the MBG model are summarised in Supplementary Table 8. Nominal range is the distance (in km) at which spatial correlation has declined to about 0.1. Estimated random effects for the environmental suitability covariate are displayed in Supplementary Figure 10.

**Supplementary Table 8. Parameter estimates from in-sample onchocerciasis MBG model.**

GP: Gaussian process. i.i.d.: Independent and identically distributed random effects. RW2: Second-order random walk model.

| **Parameter** | **Median (95% UI)** |
| --- | --- |
| Intercept | -3.656 (-4.214, -3.099) |
| GP nominal range | 189.6 (176.7, 207.1) |
| GP nominal variance | 3.177 (2.892, 3.568) |
| Nugget precision (i.i.d.) | 0.719 (0.683, 0.742) |
| Country precision (i.i.d.) | 0.505 (0.268, 0.992) |
| Environmental suitability precision (RW2) | 32.269 (5.731, 125.009) |
| Covariate coefficients |  |
| MDA (allmda) | -1.106 (-1.136, -1.077) |
| Diurnal temperature range (crutsdtr) | -0.363 (-0.537, -0.199) |
| Temperature (crutstmp) | -0.172 (-0.325, -0.018) |
| Wet day frequency (crutswet) | -0.163 (-0.315, -0.012) |
| Distance to rivers >25 m wide (distrivers25m) | -0.021 (-0.070, 0.028) |
| Enhanced Vegetation Index (evi_v6) | 0.192 (0.129, 0.256) |
| Precipitation (mswep) | 0.137 (0.057, 0.217) |
| Soil: Probability of bedrock exposure (sgbdrlog) | -0.063 (-0.119, -0.007) |
| Soil: Bulk density (200 cm depth) (sgbldfie) | 0.100 (0.038, 0.163) |
| Soil: Clay content mass fraction (200 cm depth) (sgclyppt) | -0.078 (-0.157, 0.002) |
| Soil: Coarse fragments (200 cm depth) (sgcrfvol) | 0.077 (0.008, 0.146) |
| Slope (slope) | 0.008 (-0.021, 0.038) |
| Tasseled cap brightness (tcb_v6) | -0.333 (-0.404, -0.262) |
| Population (worldpop) | -0.014 (-0.037, 0.010) |
| River width (river_size) | 0.072 (0.039, 0.106) |

Supplementary Figure 10. Estimated random effects for environmental suitability.

Random effect estimates (mean and 95% UI) are shown for the environmental suitability layer, fit within INLA using a second-order random walk (RW2) model with standard-normalised suitability values (x-axis) grouped into 25 bins by quantile; y-axis values indicate effects in logit space.

### 5.3.9 *Loa loa* endemicity

The co-occurrence of *O. volvulus* and the filarial nematode *Loa loa* complicates onchocerciasis control in some parts of central Africa,(86,87) due to potentially severe complications from ivermectin treatment in individuals with high *L. loa* microfilariae loads.(88,89) Previous modelling studies have estimated the scale of co-infections between *O. volvulus* and *L. loa* and have suggested a substantial burden of co-infection or populations at risk.(90,91) We complemented these studies by calculating the mean number of onchocerciasis cases estimated by our geospatial model in 2018, in areas considered meso- or hyper-endemic for loiasis according to endemicity classifications from ESPEN. We obtained data from ESPEN for loiasis endemicity in 2015 at the level of Implementation Units and combined this with the WorldPop population raster and our onchocerciasis mean prevalence estimates, deriving a mean estimate of 7 146 618 onchocerciasis cases in loiasis-endemic areas (excluding hypo-endemic regions) in 2018.

## **5.4 Model validation**

### 5.4.1 Metrics of predictive validity

In order to assess the predictive validity of our estimates, we validated our models using spatially stratified five-fold out-of-sample cross-validation. To construct each spatial fold, we used a modified bi-tree algorithm to spatially aggregate datapoints. This algorithm recursively partitions two-dimensional space, alternating between horizontal and vertical splits on the weighted data sample size medians, until the data contained within each spatial partition are of a similar sample size. The depth of recursive partitioning is constrained by the target sample size within a partition and the minimum number of clusters or pseudo-clusters allowed within each spatial partition (in this case, a minimum sample size of 500 was used). These spatial partitions are then allocated to one of five folds for cross-validation. Temporal partitioning was unstructured (random).

For validation, each geostatistical model was run five times, each time holding out data from one of the folds. A set of out-of-sample predictions were generated by sampling from the posterior predictive distribution for each held-out datapoint. A full suite of out-of-sample predictions over the entire dataset was calculated by combining the out-of-sample predictions from the five cross-validation runs. Using these out-of-sample predictions, we computed mean error (bias), mean absolute error, 95% coverage of the predictive intervals (the proportion of observed out-of-sample data that fall within the predicted 95% uncertainty intervals), root-mean squared error (RMSE, which summarises error variance), and the correlation of predicted versus observed prevalence at the level of individual datapoints. A scatterplot of reported prevalence versus mean out-of-sample predictions is provided in Supplementary Figure 11, and validation metrics are summarised in Supplementary Table 9.

In addition to performing cross-validation, we also evaluated over-dispersion in the model input data by performing posterior predictive checks. Briefly, a binomial count was simulated from each of 1,000 model draws of predictions for each input data row. The distribution of predictions provided a good approximation of the observed distribution, suggesting that the model is adequately addressing possible over-dispersion in the data.


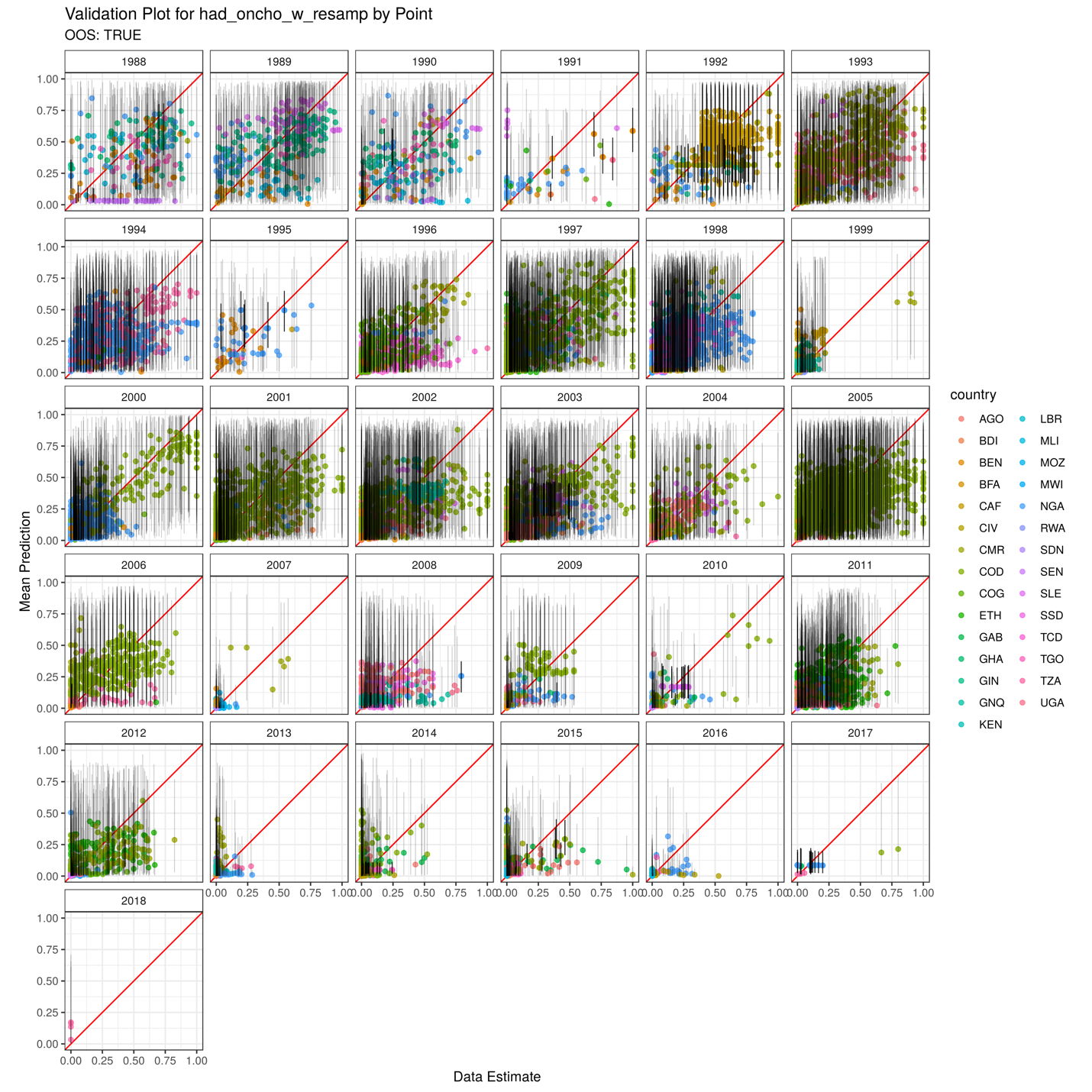


Supplementary Figure 11. Model validation scatterplots for Africa and Yemen.

Reported prevalence versus mean out-of-sample predictions for individual datapoints, by year and country. Vertical bars represent 95% UI; red lines indicate equivalence.

**Supplementary Table 9. Out-of-sample validation metrics at the level of individual datapoints, from five-fold cross-validation**

Out-of-sample performance was aggregated over 1988–2018 and is also provided for individual model years. Values were computed in prevalence space. N observations: Number of data rows in the full dataset from a given year. Mean abs. error: Mean absolute error. 95% cov.: 95% coverage. Corr.: Correlation.

| **Year** | **N Observations** | **Mean error** | **Mean abs. error** | **95% cov.** | **RMSE** | **Corr.** |
| --- | --- | --- | --- | --- | --- | --- |
| 1988 | 216 | 0.158 | 0.257 | 0.837 | 0.298 | 0.298 |
| 1989 | 310 | 0.017 | 0.155 | 0.934 | 0.205 | 0.645 |
| 1990 | 232 | -0.081 | 0.171 | 0.919 | 0.209 | 0.565 |
| 1991 | 58 | 0.159 | 0.317 | 0.639 | 0.407 | -0.003 |
| 1992 | 983 | 0.040 | 0.130 | 0.961 | 0.174 | 0.658 |
| 1993 | 866 | -0.005 | 0.175 | 0.920 | 0.233 | 0.554 |
| 1994 | 770 | 0.060 | 0.159 | 0.941 | 0.210 | 0.359 |
| 1995 | 58 | -0.037 | 0.124 | 0.908 | 0.161 | 0.500 |
| 1996 | 1 104 | 0.021 | 0.093 | 0.967 | 0.144 | 0.709 |
| 1997 | 1 148 | -0.008 | 0.138 | 0.959 | 0.189 | 0.745 |
| 1998 | 1 620 | -0.023 | 0.109 | 0.970 | 0.152 | 0.615 |
| 1999 | 370 | -0.052 | 0.074 | 0.942 | 0.126 | 0.575 |
| 2000 | 958 | -0.026 | 0.089 | 0.978 | 0.123 | 0.814 |
| 2001 | 1 163 | -0.010 | 0.112 | 0.954 | 0.163 | 0.707 |
| 2002 | 885 | 0.012 | 0.147 | 0.913 | 0.200 | 0.547 |
| 2003 | 1 082 | 0.017 | 0.119 | 0.953 | 0.173 | 0.615 |
| 2004 | 397 | 0.009 | 0.113 | 0.930 | 0.153 | 0.658 |
| 2005 | 1 930 | 0.005 | 0.144 | 0.965 | 0.188 | 0.533 |
| 2006 | 383 | -0.002 | 0.104 | 0.727 | 0.170 | 0.611 |
| 2007 | 117 | -0.017 | 0.046 | 0.899 | 0.083 | 0.631 |
| 2008 | 367 | 0.046 | 0.112 | 0.902 | 0.195 | 0.666 |
| 2009 | 193 | -0.010 | 0.055 | 0.816 | 0.092 | 0.566 |
| 2010 | 133 | 0.020 | 0.066 | 0.904 | 0.097 | 0.722 |
| 2011 | 764 | 0.009 | 0.097 | 0.903 | 0.140 | 0.620 |
| 2012 | 543 | 0.014 | 0.063 | 0.934 | 0.115 | 0.659 |
| 2013 | 477 | -0.014 | 0.021 | 0.965 | 0.046 | 0.207 |
| 2014 | 397 | -0.035 | 0.051 | 0.914 | 0.096 | 0.344 |
| 2015 | 472 | -0.043 | 0.077 | 0.853 | 0.129 | 0.182 |
| 2016 | 96 | 0.004 | 0.033 | 0.955 | 0.075 | 0.138 |
| 2017 | 20 | 0.062 | 0.096 | 0.995 | 0.189 | 0.881 |
| 2018 | 4 | -0.138 | 0.138 | 0.524 | 0.145 | NA |
| 1988–2018 | 18 116 | 0.003 | 0.111 | 0.924 | 0.168 | 0.706 |

# **6.0 Supplementary references**

1. ESPEN. ESPEN. Available from: https://www.espen.org/

2. WHO | Geographical distribution of onchocerciasis. WHO. World Health Organization; Available from: https://www.who.int/onchocerciasis/distribution/en/

3. Remme JHF. The African programme for onchocerciasis control: preparing to launch. Parasitol Today. 1995;11(11):403–6.

4. Ngoumou P, Walsh JF, Mace JM. A rapid mapping technique for the prevalence and distribution of onchocerciasis: a Cameroon case study. Ann Trop Med Parasitol. 1994;88(5):463–74.

5. Ngoumou P, Walsh JF, Blindness WP for the P of, Diseases UBSP for R and T in T. A manual for rapid epidemiological mapping of onchocerciasis. 1993; Available from: https://apps.who.int/iris/handle/10665/59537

6. Noma M, Nwoke BEB, Nutall I, Tambala PA, Enyong P, Namsenmo A, et al. Rapid epidemiological mapping of onchocerciasis (REMO): its application by the African Programme for Onchocerciasis Control (APOC). Ann Trop Med Parasitol. 2002;96 Suppl 1:S29-39.

7. Tekle AH, Zouré HGM, Noma M, Boussinesq M, Coffeng LE, Stolk WA, et al. Progress towards onchocerciasis elimination in the participating countries of the African Programme for Onchocerciasis Control: epidemiological evaluation results. Infect Dis Poverty. 2016;5(1):66.

8. WHO | African Programme for Onchocerciasis Control: progress report, 2013–2014. Wkly Epidemiol Rec. 2014;89(49):551–60.

9. ENVISION. Tanzania Work Plan, FY2018. ENVISION; 2017. Available from: https://www.ntdenvision.org/sites/default/files/docs/tanzania_work_planfy18.pdf

10. de Smet E, Metanmo S, Mbelesso P, Kemata B, Siewe Fodjo JN, Boumédiène F, et al. Focus of ongoing onchocerciasis transmission close to Bangui, Central African Republic. Pathogens. 2020;9(5):337.

11. Bof JCM, Maketa V, Bakajika DK, Ntumba F, Mpunga D, Murdoch ME, et al. Onchocerciasis control in the Democratic Republic of Congo (DRC): challenges in a post-war environment. Trop Med Int Health. 2015;20(1):48–62.

12. Makenga Bof JC, Ntumba Tshitoka F, Muteba D, Mansiangi P, Coppieters Y. Review of the National Program for Onchocerciasis Control in the Democratic Republic of the Congo. Trop Med Infect Dis. 2019;4(2):92.

13. Samuel A, Belay T, Yehalaw D, Taha M, Zemene E, Zeynudin A. Impact of six years community directed treatment with ivermectin in the control of onchocerciasis, Western Ethiopia. PLOS ONE. 2016;11(3):e0141029.

14. Gemade EII, Jiya JY, Nwoke BEB, Ogunba EO, Edeghere H, Akoh JI, et al. Human onchocerciasis: current assessment of the disease burden in Nigeria by rapid epidemiological mapping. Ann Trop Med Parasitol. 1998;92(sup1):S79–83.

15. Republic of Rwanda Ministry of Health. Neglected Tropical Diseases Strategic Plan 2019-2024. Republic of Rwanda Ministry of Health; 2019. Available from: http://rbc.gov.rw/fileadmin/user_upload/guide2019/guide2019/RWANDA%20NTD%20STRATEGIC%20PLAN%202019-2024.pdf

16. Laggo MCL, Chane F. Onchocerciasis control in South Sudan. South Sudan Med J. 2011;4(3):61–2.

17. World Bank. Chad, Cameroon - Petroleum Development and Pipeline Project: environmental assessment. Washington, D.C.: World Bank; 1999. Available from: http://documents.banquemondiale.org/curated/fr/863021468769828252/pdf/multi-page.pdf

18. Katabarwa MN, Lakwo T, Habomugisha P, Unnasch TR, Garms R, Hudson-Davis L, et al. After 70 years of fighting an age-old scourge, onchocerciasis in Uganda, the end is in sight. Int Health. 2018;10(suppl_1):i79–88.

19. ESPEN Portal. Available from: https://admin.espen.afro.who.int/

20. WHO | Global Programme to Eliminate Lymphatic Filariasis. WHO. World Health Organization; Available from: http://www.who.int/lymphatic_filariasis/elimination-programme/en/

21. Graham MH. Confronting Multicollinearity in Ecological Multiple Regression. Ecology. 2003;84(11):2809–15.

22. Faraway JJ. Linear Models with R. Boca Raton: CRC Press; 2005.

23. Prost A, Thylefors B, Pairault C. Methods of mass epidemiological evaluation of onchocerciasis: their utilization in a vector control programme. Expert Comm Epidemiol Onchocerciasis. 1975;20.

24. O’Hanlon SJ, Slater HC, Cheke RA, Boatin BA, Coffeng LE, Pion SDS, et al. Model-based geostatistical mapping of the prevalence of Onchocerca volvulus in West Africa. PLoS Negl Trop Dis. 2016;10(1):e0004328.

25. World Health Organization. Supplemental Guidelines for Rapid Epidemiological Mapping of Onchocerciasis ( REMO). Geneva, Switzerland: World Health Organization; 1995. Report No.: Document TDR/TDF/ONCHO/95.1. Available from: https://www.who.int/tdr/publications/tdr-research-publications/oncho-guidelines/en/

26. Zouré HG, Noma M, Tekle AH, Amazigo UV, Diggle PJ, Giorgi E, et al. The geographic distribution of onchocerciasis in the 20 participating countries of the African Programme for Onchocerciasis Control: (2) pre-control endemicity levels and estimated number infected. Parasit Vectors. 2014;7(1):326.

27. Gallin M, Adams A, Kruppa TF, Gbaguidi EA, Massougbodji A, Sadeler BC, et al. Epidemiological studies of onchocerciasis in southern Benin. Trop Med Parasitol Off Organ Dtsch Tropenmedizinische Ges Dtsch Ges Tech Zusammenarbeit GTZ. 1993;44(2):69–74.

28. Boussinesq M, Chippaux JP, Ernould JC, Quillevere D, Prod’hon J. Effect of repeated treatments with ivermectin on the incidence of onchocerciasis in northern Cameroon. Am J Trop Med Hyg. 1995;53(1):63–7.

29. Mendoza Aldana J, Piechulek H, Maguire J. Forest onchocerciasis in Cameroon: its distribution and implications for selection of communities for control programmes. Ann Trop Med Parasitol. 1997;91(1):79–86.

30. Katabarwa MN, Eyamba A, Nwane P, Enyong P, Kamgno J, Kueté T, et al. Fifteen years of annual mass treatment of onchocerciasis with ivermectin have not interrupted transmission in the west region of Cameroon. J Parasitol Res. 2013;2013. Available from: https://www.ncbi.nlm.nih.gov/pmc/articles/PMC3652197/

31. Kamgno J, Boussinesq M. Hyperendemic loaiasis in the Tikar plain, shrub savanna region of Cameroon. Bull Soc Pathol Exot 1990. 2001;94(4):342–6.

32. Katabarwa MN, Eyamba A, Chouaibou M, Enyong P, Kuété T, Yaya S, et al. Does onchocerciasis transmission take place in hypoendemic areas? a study from the North Region of Cameroon. Trop Med Int Health. 2010;15(5):645–52.

33. Katabarwa MN, Eyamba A, Nwane P, Enyong P, Yaya S, Baldiagaï J, et al. Seventeen years of annual distribution of ivermectin has not interrupted onchocerciasis transmission in North Region, Cameroon. Am J Trop Med Hyg. 2011;85(6):1041–9.

34. Kamga HLF, Shey DN, Assob JCN, Njunda AL, Nde Fon P, Njem PK. Prevalence of onchocerciasis in the Fundong Health District, Cameroon after 6 years of continuous community-directed treatment with ivermectin. Pan Afr Med J. 2011;10. Available from: https://www.ncbi.nlm.nih.gov/pmc/articles/PMC3240934/

35. Wanji S, Kengne-Ouafo JA, Esum ME, Chounna PWN, Adzemye BF, Eyong JEE, et al. Relationship between oral declaration on adherence to ivermectin treatment and parasitological indicators of onchocerciasis in an area of persistent transmission despite a decade of mass drug administration in Cameroon. Parasit Vectors. 2015;8. Available from: https://www.ncbi.nlm.nih.gov/pmc/articles/PMC4696282/

36. Kamga GR, Dissak-Delon FN, Nana-Djeunga HC, Biholong BD, Ghogomu SM, Souopgui J, et al. Important progress towards elimination of onchocerciasis in the West Region of Cameroon. Parasit Vectors. 2017;10. Available from: https://www.ncbi.nlm.nih.gov/pmc/articles/PMC5543544/

37. Kamga GR, Dissak-Delon FN, Nana-Djeunga HC, Biholong BD, Mbigha-Ghogomu S, Souopgui J, et al. Still mesoendemic onchocerciasis in two Cameroonian community-directed treatment with ivermectin projects despite more than 15 years of mass treatment. Parasit Vectors. 2016;9(1):581.

38. Bakajika D, Senyonjo L, Enyong P, Oye J, Biholong B, Elhassan E, et al. On-going transmission of human onchocerciasis in the Massangam health district in the West Region of Cameroon: Better understanding transmission dynamics to inform changes in programmatic interventions. PLoS Negl Trop Dis. 2018;12(11):e0006904.

39. Katabarwa M, Eyamba A, Habomugisha P, Lakwo T, Ekobo S, Kamgno J, et al. After a decade of annual dose mass ivermectin treatment in Cameroon and Uganda, onchocerciasis transmission continues. Trop Med Int Health TM IH. 2008;13(9):1196–203.

40. Kennedy MH, Bertocchi I, Hopkins AD, Meredith SE. The effect of 5 years of annual treatment with ivermectin (Mectizan) on the prevalence and morbidity of onchocerciasis in the village of Gami in the Central African Republic. Ann Trop Med Parasitol. 2002;96(3):297–307.

41. Taye A, Gebre-Michael T, Taticheff S. Onchocerciasis in Gilgel Ghibe River Valley southwest Ethiopia. East Afr Med J. 2000;77(2):116–20.

42. Mengistu G, Balcha F, Britton S. Co-infection of Onchocerca volvulus and intestinal helminths in indigenous and migrant farmers in southwest Ethiopia. Ethiop Med J. 2002;40(1):19–27.

43. Legesse M, Balcha F, Erko B. Status of onchocerciasis in Teppi area, Southwestern Ethiopia, after four years of annual community-directed treatment with ivermectin. Ethiop J Health Dev. 2010;24(1). Available from: https://www.ajol.info/index.php/ejhd/article/view/62945

44. Dori GU, Belay T, Belete H, Panicker KN, Hailu A. Parasitological and clinico-epidemiological features of onchocerciasis in West Wellega, Ethiopia. J Parasit Dis Off Organ Indian Soc Parasitol. 2012;36(1):10–8.

45. Dana D, Debalke S, Mekonnen Z, Kassahun W, Suleman S, Getahun K, et al. A community-based cross-sectional study of the epidemiology of onchocerciasis in unmapped villages for community directed treatment with ivermectin in Jimma Zone, southwestern Ethiopia. BMC Public Health. 2015;15:595.

46. Fobi G, Mourou Mbina JR, Ozoh G, Kombila M, Agaya C, Olinga Olinga JM, et al. Onchocerciasis in the area of Lastourville, Gabon. Clinical and entomological aspects. Bull Soc Pathol Exot 1990. 2006;99(4):269–71.

47. Anosike JC, Onwuliri CO. Studies on filariasis in Bauchi State, Nigeria. II. The prevalence of human filariasis in Darazo Local Government area. Appl Parasitol. 1994;35(4):242–50.

48. Osue HO, Inabo HI, Yakubu SE, Audu PA, Galadima M, Odama LE, et al. Impact of eighteen-year varied compliance to onchocerciasis treatment with ivermectin in sentinel savannah agrarian communities in Kaduna State of Nigeria. ISRN Parasitol. 2013;2013:960168.

49. Anosike JC, Celestine null, Onwuliri OE, Onwuliri VA. The prevalence, intensity and clinical manifestations of Onchocerca volvulus infection in Toro local government area of Bauchi State, Nigeria. Int J Hyg Environ Health. 2001;203(5–6):459–64.

50. Umeh RE. The causes and profile of visual loss in an onchocerciasis-endemic forest-savanna zone in Nigeria. Ophthalmic Epidemiol. 1999;6(4):303–15.

51. Nmorsi OPG, Oladokun I a. A, Egwunyenga OA, Oseha E. Eye lesions and onchocerciasis in a rural farm settlement in Delta state, Nigeria. Southeast Asian J Trop Med Public Health. 2002;33(1):28–32.

52. Okoye IC, Onwuliri CO. Epidemiology and psycho-social aspects of onchocercal skin diseases in northeastern Nigeria. Filaria J. 2007;6:15.

53. Rebecca SN, Akinboye DO, Abdulazeez AA. Onchocerciasis and plasmodiasis: concurrent infection in Garaha-Dutse community, Adamawa State Nigeria. Biomed Res. 2008;19(2). Available from: https://www.alliedacademies.org/abstract/onchocerciasis-and-plasmodiasis-concurrent-infectionrnin-garahadutse-community-adamawa-state-nigeria-802.html

54. Emukah E, Nimzing J, Ezeabikwa M, Obiezu J, Okpala N, Miri E, et al. Impact assessment of repeated annual ivermectin on ocular and clinical onchocerciasis 14 years of annual mass drug administration in eight sentinel villages, southeast Nigeria. Am J Trop Med Hyg. 2010;83(Suppl 5):22.

55. Akinbo FO, Okaka CE. Hyperendemicity of onchocerciasis in Ovia Northeast Local Government Area, Edo State, Nigeria. East Afr J Public Health. 2010;7(1):84–6.

56. Akinboye, D.O, Okwong, E, N A, Agbolade FO, et al. Onchocerciasis among inhabitants of Ibarapa local government community of Oyo state, Nigeria. Biomed Res. 2010;21(2). Available from: https://www.alliedacademies.org/abstract/onchocerciasis-among-inhabitants-of-ibarapa-local-governmentrncommunity-of-oyo-state-nigeria-1287.html

57. Uttah E, Ibeh DC. Multiple filarial species microfilaraemia: a comparative study of areas with endemic and sporadic onchocerciasis. J Vector Borne Dis. 2011;48(4):197–204.

58. Evans DS, Alphonsus K, Umaru J, Eigege A, Miri E, Mafuyai H, et al. Status of onchocerciasis transmission after more than a decade of mass drug administration for onchocerciasis and lymphatic filariasis elimination in Central Nigeria: challenges in coordinating the Stop MDA Decision. PLoS Negl Trop Dis. 2014;8(9). Available from: https://www.ncbi.nlm.nih.gov/pmc/articles/PMC4169246/

59. Eyo J, Onyishi G, Ugokwe C. Rapid epidemiological assessment of onchocerciasis in a tropical semi-urban community, enugu state, Nigeria. Iran J Parasitol. 2013;8(1):145–51.

60. Koroma JB, Sesay S, Conteh A, Koudou B, Paye J, Bah M, et al. Impact of five annual rounds of mass drug administration with ivermectin on onchocerciasis in Sierra Leone. Infect Dis Poverty. 2018;7(1):30.

61. Dogba PK, Komi BT. The evaluation of endemic onchocerciasis in the Amou prefecture (Togo). Bull Soc Pathol Exot 1990. 1994;87(2):110–1.

62. Golden A, Faulx D, Kalnoky M, Stevens E, Yokobe L, Peck R, et al. Analysis of age-dependent trends in Ov16 IgG4 seroprevalence to onchocerciasis. Parasit Vectors. 2016;9(1):338.

63. Komlan K, Vossberg PS, Gantin RG, Solim T, Korbmacher F, Banla M, et al. Onchocerca volvulus infection and serological prevalence, ocular onchocerciasis and parasite transmission in northern and central Togo after decades of Simulium damnosum s.l. vector control and mass drug administration of ivermectin. PLoS Negl Trop Dis. 2018;12(3):e0006312.

64. Katabarwa MN, Lakwo T, Habomugisha P, Agunyo S, Byamukama E, Oguttu D, et al. Transmission of Onchocerca volvulus continues in Nyagak-Bondo focus of northwestern Uganda after 18 years of a single dose of annual treatment with ivermectin. Am J Trop Med Hyg. 2013;89(2):293–300.

65. Luroni LT, Gabriel M, Tukahebwa E, Onapa AW, Tinkitina B, Tukesiga E, et al. The interruption of Onchocerca volvulus and Wuchereria bancrofti transmission by integrated chemotherapy in the Obongi focus, North Western Uganda. PloS One. 2017;12(12):e0189306.

66. Murray CJL, Callender CSKH, Kulikoff XR, Srinivasan V, Abate D, Abate KH, et al. Population and fertility by age and sex for 195 countries and territories, 1950–2017: a systematic analysis for the Global Burden of Disease Study 2017. The Lancet. 2018;392(10159):1995–2051.

67. Ramsay JO, Wickham H, Graves S, Hooker G. fda: Functional Data Analysis. 2018. Available from: https://CRAN.R-project.org/package=fda

68. Byrd RH, Lu P, Nocedal J, Zhu C. A limited memory algorithm for bound constrained optimization. SIAM J Sci Comput. 1995;16(5):1190–208.

69. R: The R Project for Statistical Computing. Available from: https://www.r-project.org/

70. Kirkwood B, Smith P, Marshall T, Prost A. Variations in the prevalence and intensity of microfilarial infections by age, sex, place and time in the area of the Onchocerciasis Control Programme. Trans R Soc Trop Med Hyg. 1983;77(6):857–61.

71. Vivas-Martínez S, Basáñez MG, Botto C, Rojas S, García M, Pacheco M, et al. Amazonian onchocerciasis: parasitological profiles by host-age, sex, and endemicity in southern Venezuela. Parasitology. 2000;121(5):513–25.

72. Anderson RM, Basáñez MG, Boussinesq M. Population biology of human onchocerciasis. Philos Trans R Soc Lond B Biol Sci. 1999;354(1384):809–26.

73. Coffeng LE, Pion SDS, O’Hanlon S, Cousens S, Abiose AO, Fischer PU, et al. Onchocerciasis: the pre-control association between prevalence of palpable nodules and skin microfilariae. PLoS Negl Trop Dis. 2013;7(4):e2168.

74. Hamley JID, Milton P, Walker M, Basáñez MG. Modelling exposure heterogeneity and density dependence in onchocerciasis using a novel individual-based transmission model, EPIONCHO-IBM: Implications for elimination and data needs. PLoS Negl Trop Dis. 2019;13(12):e0007557.

75. Gebrezgabiher G, Mekonnen Z, Yewhalaw D, Hailu A. Reaching the last mile: main challenges relating to and recommendations to accelerate onchocerciasis elimination in Africa. Infect Dis Poverty. 2019;8(1):60.

76. Vlaminck J, Fischer PU, Weil GJ. Diagnostic tools for onchocerciasis elimination programs. Trends Parasitol. 2015;31(11):571–82.

77. Turner HC, Walker M, Churcher TS, Basáñez MG. Modelling the impact of ivermectin on River Blindness and its burden of morbidity and mortality in African Savannah: EpiOncho projections. Parasit Vectors. 2014;7(1):241.

78. Remme. The global burden of onchocerciasis in 1990. Geneva, Switzerland: World Health Organization; 2004 p. 26. Available from: https://www.who.int/apoc/publications/bod_onchocerciasis1990_the_global_burden_of_onchocerciasis_in_1990.pdf

79. Unnasch TR, Golden A, Cama V, Cantey PT. Diagnostics for onchocerciasis in the era of elimination. Int Health. 2018;10(suppl_1):i20–6.

80. Golding N, Burstein R, Longbottom J, Browne AJ, Fullman N, Osgood-Zimmerman A, et al. Mapping under-5 and neonatal mortality in Africa, 2000–15: a baseline analysis for the Sustainable Development Goals. The Lancet. 2017;390(10108):2171–82.

81. Cromwell EA, Osborne JCP, Unnasch TR, Basáñez MG, Gass KM, Barbre KA, et al. Predicting the environmental suitability for onchocerciasis in Africa as an aid to elimination planning. PLoS Negl Trop Dis. 2021;15(7):e0008824.

82. Gómez‐Rubio V, Palmí‐Perales F. Multivariate posterior inference for spatial models with the integrated nested Laplace approximation. J R Stat Soc Ser C Appl Stat. 2019;68(1):199–215.

83. Lindgren F, Rue H, Lindström J. An explicit link between Gaussian fields and Gaussian Markov random fields: the stochastic partial differential equation approach. Stat Methodol Ser B. 2011;73(4):423–98.

84. Blangiardo M, Cameletti M, Baio G, Rue H. Spatial and spatio-temporal models with R-INLA. Spat Spatio-Temporal Epidemiol. 2013;7:39–55.

85. Lindgren F, Rue H. Bayesian Spatial Modelling with R- INLA. J Stat Softw. 2015;63(19):1–25.

86. Wanji S, Ndongmo WPC, Fombad FF, Kengne-Ouafo JA, Njouendou AJ, Tchounkeu YFL, et al. Impact of repeated annual community directed treatment with ivermectin on loiasis parasitological indicators in Cameroon: Implications for onchocerciasis and lymphatic filariasis elimination in areas co-endemic with Loa loa in Africa. PLoS Negl Trop Dis. 2018;12(9):e0006750.

87. Zouré HGM, Wanji S, Noma M, Amazigo UV, Diggle PJ, Tekle AH, et al. The geographic distribution of Loa loa in Africa: results of large-scale implementation of the rapid assessment procedure for loiasis (RAPLOA). PLoS Negl Trop Dis. 2011;5(6):e1210.

88. Gardon J, Gardon-Wendel N, Demanga-Ngangue, Kamgno J, Chippaux JP, Boussinesq M. Serious reactions after mass treatment of onchocerciasis with ivermectin in an area endemic for Loa loa infection. The Lancet. 1997;350(9070):18–22.

89. Boussinesq M, Gardon J, Gardon-Wendel N, Chippaux JP. Clinical picture, epidemiology and outcome of Loa-associated serious adverse events related to mass ivermectin treatment of onchocerciasis in Cameroon. Filaria J. 2003;2(1):S4.

90. Vinkeles Melchers NVS, Coffeng LE, Boussinesq M, Pedrique B, Pion SDS, Tekle AH, et al. Projected number of people with onchocerciasis–loiasis coinfection in Africa, 1995 to 2025. Clin Infect Dis. 2020;70(11):2281–9.

91. Cano J, Basáñez MG, O’Hanlon SJ, Tekle AH, Wanji S, Zouré HG, et al. Identifying co-endemic areas for major filarial infections in sub-Saharan Africa: seeking synergies and preventing severe adverse events during mass drug administration campaigns. Parasit Vectors. 2018;11(1):70.
